# Measurement of the differential cross section for top quark pair production in pp collisions at $\sqrt{s} = 8\,\text {TeV}$

**DOI:** 10.1140/epjc/s10052-015-3709-x

**Published:** 2015-11-20

**Authors:** V. Khachatryan, A. M. Sirunyan, A. Tumasyan, W. Adam, T. Bergauer, M. Dragicevic, J. Erö, M. Friedl, R. Frühwirth, V. M. Ghete, C. Hartl, N. Hörmann, J. Hrubec, M. Jeitler, W. Kiesenhofer, V. Knünz, M. Krammer, I. Krätschmer, D. Liko, I. Mikulec, D. Rabady, B. Rahbaran, H. Rohringer, R. Schöfbeck, J. Strauss, W. Treberer-Treberspurg, W. Waltenberger, C.-E. Wulz, V. Mossolov, N. Shumeiko, J. Suarez Gonzalez, S. Alderweireldt, M. Bansal, S. Bansal, T. Cornelis, E. A. De Wolf, X. Janssen, A. Knutsson, S. Luyckx, S. Ochesanu, R. Rougny, M. Van De Klundert, H. Van Haevermaet, P. Van Mechelen, N. Van Remortel, A. Van Spilbeeck, F. Blekman, S. Blyweert, J. D’Hondt, N. Daci, N. Heracleous, J. Keaveney, S. Lowette, M. Maes, A. Olbrechts, Q. Python, D. Strom, S. Tavernier, W. Van Doninck, P. Van Mulders, G. P. Van Onsem, I. Villella, C. Caillol, B. Clerbaux, G. De Lentdecker, D. Dobur, L. Favart, A. P. R. Gay, A. Grebenyuk, A. Léonard, A. Mohammadi, L. Perniè, T. Reis, T. Seva, L. Thomas, C. Vander Velde, P. Vanlaer, J. Wang, F. Zenoni, V. Adler, K. Beernaert, L. Benucci, A. Cimmino, S. Costantini, S. Crucy, S. Dildick, A. Fagot, G. Garcia, J. Mccartin, A. A. Ocampo Rios, D. Ryckbosch, S. Salva Diblen, M. Sigamani, N. Strobbe, F. Thyssen, M. Tytgat, E. Yazgan, N. Zaganidis, S. Basegmez, C. Beluffi, G. Bruno, R. Castello, A. Caudron, L. Ceard, G. G. Da Silveira, C. Delaere, T. du Pree, D. Favart, L. Forthomme, A. Giammanco, J. Hollar, A. Jafari, P. Jez, M. Komm, V. Lemaitre, C. Nuttens, D. Pagano, L. Perrini, A. Pin, K. Piotrzkowski, A. Popov, L. Quertenmont, M. Selvaggi, M. Vidal Marono, J. M. Vizan Garcia, N. Beliy, T. Caebergs, E. Daubie, G. H. Hammad, W. L. Aldá Júnior, G. A. Alves, L. Brito, M. Correa Martins Junior, T. Dos Reis Martins, C. Mora Herrera, M. E. Pol, W. Carvalho, J. Chinellato, A. Custódio, E. M. Da Costa, D. De Jesus Damiao, C. De Oliveira Martins, S. Fonseca De Souza, H. Malbouisson, D. Matos Figueiredo, L. Mundim, H. Nogima, W. L. Prado Da Silva, J. Santaolalla, A. Santoro, A. Sznajder, E. J. Tonelli Manganote, A. Vilela Pereira, C. A. Bernardes, S. Dogra, T. R. Fernandez Perez Tomei, E. M. Gregores, P. G. Mercadante, S. F. Novaes, Sandra S. Padula, A. Aleksandrov, V. Genchev, P. Iaydjiev, A. Marinov, S. Piperov, M. Rodozov, G. Sultanov, M. Vutova, A. Dimitrov, I. Glushkov, R. Hadjiiska, V. Kozhuharov, L. Litov, B. Pavlov, P. Petkov, J. G. Bian, G. M. Chen, H. S. Chen, M. Chen, R. Du, C. H. Jiang, R. Plestina, F. Romeo, J. Tao, Z. Wang, C. Asawatangtrakuldee, Y. Ban, Q. Li, S. Liu, Y. Mao, S. J. Qian, D. Wang, W. Zou, C. Avila, L. F. Chaparro Sierra, C. Florez, J. P. Gomez, B. Gomez Moreno, J. C. Sanabria, N. Godinovic, D. Lelas, D. Polic, I. Puljak, Z. Antunovic, M. Kovac, V. Brigljevic, K. Kadija, J. Luetic, D. Mekterovic, L. Sudic, A. Attikis, G. Mavromanolakis, J. Mousa, C. Nicolaou, F. Ptochos, P. A. Razis, M. Bodlak, M. Finger, M. Finger, Y. Assran, A. Ellithi Kamel, M. A. Mahmoud, A. Radi, M. Kadastik, M. Murumaa, M. Raidal, A. Tiko, P. Eerola, G. Fedi, M. Voutilainen, J. Härkönen, V. Karimäki, R. Kinnunen, M. J. Kortelainen, T. Lampén, K. Lassila-Perini, S. Lehti, T. Lindén, P. Luukka, T. Mäenpää, T. Peltola, E. Tuominen, J. Tuominiemi, E. Tuovinen, L. Wendland, J. Talvitie, T. Tuuva, M. Besancon, F. Couderc, M. Dejardin, D. Denegri, B. Fabbro, J. L. Faure, C. Favaro, F. Ferri, S. Ganjour, A. Givernaud, P. Gras, G. Hamel de Monchenault, P. Jarry, E. Locci, J. Malcles, J. Rander, A. Rosowsky, M. Titov, S. Baffioni, F. Beaudette, P. Busson, C. Charlot, T. Dahms, M. Dalchenko, L. Dobrzynski, N. Filipovic, A. Florent, R. Granier de Cassagnac, L. Mastrolorenzo, P. Miné, C. Mironov, I. N. Naranjo, M. Nguyen, C. Ochando, P. Paganini, S. Regnard, R. Salerno, J. B. Sauvan, Y. Sirois, C. Veelken, Y. Yilmaz, A. Zabi, J.-L. Agram, J. Andrea, A. Aubin, D. Bloch, J.-M. Brom, E. C. Chabert, C. Collard, E. Conte, J.-C. Fontaine, D. Gelé, U. Goerlach, C. Goetzmann, A.-C. Le Bihan, P. Van Hove, S. Gadrat, S. Beauceron, N. Beaupere, G. Boudoul, E. Bouvier, S. Brochet, C. A. Carrillo Montoya, J. Chasserat, R. Chierici, D. Contardo, P. Depasse, H. El Mamouni, J. Fan, J. Fay, S. Gascon, M. Gouzevitch, B. Ille, T. Kurca, M. Lethuillier, L. Mirabito, S. Perries, J. D. Ruiz Alvarez, D. Sabes, L. Sgandurra, V. Sordini, M. Vander Donckt, P. Verdier, S. Viret, H. Xiao, Z. Tsamalaidze, C. Autermann, S. Beranek, M. Bontenackels, M. Edelhoff, L. Feld, O. Hindrichs, K. Klein, A. Ostapchuk, A. Perieanu, F. Raupach, J. Sammet, S. Schael, H. Weber, B. Wittmer, V. Zhukov, M. Ata, M. Brodski, E. Dietz-Laursonn, D. Duchardt, M. Erdmann, R. Fischer, A. Güth, T. Hebbeker, C. Heidemann, K. Hoepfner, D. Klingebiel, S. Knutzen, P. Kreuzer, M. Merschmeyer, A. Meyer, P. Millet, M. Olschewski, K. Padeken, P. Papacz, H. Reithler, S. A. Schmitz, L. Sonnenschein, D. Teyssier, S. Thüer, M. Weber, V. Cherepanov, Y. Erdogan, G. Flügge, H. Geenen, M. Geisler, W. Haj Ahmad, A. Heister, F. Hoehle, B. Kargoll, T. Kress, Y. Kuessel, A. Künsken, J. Lingemann, A. Nowack, I. M. Nugent, L. Perchalla, O. Pooth, A. Stahl, I. Asin, N. Bartosik, J. Behr, W. Behrenhoff, U. Behrens, A. J. Bell, M. Bergholz, A. Bethani, K. Borras, A. Burgmeier, A. Cakir, L. Calligaris, A. Campbell, S. Choudhury, F. Costanza, C. Diez Pardos, S. Dooling, T. Dorland, G. Eckerlin, D. Eckstein, T. Eichhorn, G. Flucke, J. Garay Garcia, A. Geiser, P. Gunnellini, J. Hauk, M. Hempel, D. Horton, H. Jung, A. Kalogeropoulos, M. Kasemann, P. Katsas, J. Kieseler, C. Kleinwort, D. Krücker, W. Lange, J. Leonard, K. Lipka, A. Lobanov, W. Lohmann, B. Lutz, R. Mankel, I. Marfin, I.-A. Melzer-Pellmann, A. B. Meyer, G. Mittag, J. Mnich, A. Mussgiller, S. Naumann-Emme, A. Nayak, O. Novgorodova, E. Ntomari, H. Perrey, D. Pitzl, R. Placakyte, A. Raspereza, P. M. Ribeiro Cipriano, B. Roland, E. Ron, M. Ö. Sahin, J. Salfeld-Nebgen, P. Saxena, R. Schmidt, T. Schoerner-Sadenius, M. Schröder, C. Seitz, S. Spannagel, A. D. R. Vargas Trevino, R. Walsh, C. Wissing, M. Aldaya Martin, V. Blobel, M. Centis Vignali, A. R. Draeger, J. Erfle, E. Garutti, K. Goebel, M. Görner, J. Haller, M. Hoffmann, R. S. Höing, H. Kirschenmann, R. Klanner, R. Kogler, J. Lange, T. Lapsien, T. Lenz, I. Marchesini, J. Ott, T. Peiffer, N. Pietsch, J. Poehlsen, T. Poehlsen, D. Rathjens, C. Sander, H. Schettler, P. Schleper, E. Schlieckau, A. Schmidt, M. Seidel, V. Sola, H. Stadie, G. Steinbrück, D. Troendle, E. Usai, L. Vanelderen, A. Vanhoefer, C. Barth, C. Baus, J. Berger, C. Böser, E. Butz, T. Chwalek, W. De Boer, A. Descroix, A. Dierlamm, M. Feindt, F. Frensch, M. Giffels, F. Hartmann, T. Hauth, U. Husemann, I. Katkov, A. Kornmayer, E. Kuznetsova, P. Lobelle Pardo, M. U. Mozer, Th. Müller, A. Nürnberg, G. Quast, K. Rabbertz, F. Ratnikov, S. Röcker, H. J. Simonis, F. M. Stober, R. Ulrich, J. Wagner-Kuhr, S. Wayand, T. Weiler, R. Wolf, G. Anagnostou, G. Daskalakis, T. Geralis, V. A. Giakoumopoulou, A. Kyriakis, D. Loukas, A. Markou, C. Markou, A. Psallidas, I. Topsis-Giotis, A. Agapitos, S. Kesisoglou, A. Panagiotou, N. Saoulidou, E. Stiliaris, X. Aslanoglou, I. Evangelou, G. Flouris, C. Foudas, P. Kokkas, N. Manthos, I. Papadopoulos, E. Paradas, G. Bencze, C. Hajdu, P. Hidas, D. Horvath, F. Sikler, V. Veszpremi, G. Vesztergombi, A. J. Zsigmond, N. Beni, S. Czellar, J. Karancsi, J. Molnar, J. Palinkas, Z. Szillasi, A. Makovec, P. Raics, Z. L. Trocsanyi, B. Ujvari, S. K. Swain, S. B. Beri, V. Bhatnagar, R. Gupta, U. Bhawandeep, A. K. Kalsi, M. Kaur, R. Kumar, M. Mittal, N. Nishu, J. B. Singh, Ashok Kumar, Arun Kumar, S. Ahuja, A. Bhardwaj, B. C. Choudhary, A. Kumar, S. Malhotra, M. Naimuddin, K. Ranjan, V. Sharma, S. Banerjee, S. Bhattacharya, K. Chatterjee, S. Dutta, B. Gomber, Sa. Jain, Sh. Jain, R. Khurana, A. Modak, S. Mukherjee, D. Roy, S. Sarkar, M. Sharan, A. Abdulsalam, D. Dutta, S. Kailas, V. Kumar, A. K. Mohanty, L. M. Pant, P. Shukla, A. Topkar, T. Aziz, S. Banerjee, S. Bhowmik, R. M. Chatterjee, R. K. Dewanjee, S. Dugad, S. Ganguly, S. Ghosh, M. Guchait, A. Gurtu, G. Kole, S. Kumar, M. Maity, G. Majumder, K. Mazumdar, G. B. Mohanty, B. Parida, K. Sudhakar, N. Wickramage, H. Bakhshiansohi, H. Behnamian, S. M. Etesami, A. Fahim, R. Goldouzian, M. Khakzad, M. Mohammadi Najafabadi, M. Naseri, S. Paktinat Mehdiabadi, F. Rezaei Hosseinabadi, B. Safarzadeh, M. Zeinali, M. Felcini, M. Grunewald, M. Abbrescia, C. Calabria, S. S. Chhibra, A. Colaleo, D. Creanza, N. De Filippis, M. De Palma, L. Fiore, G. Iaselli, G. Maggi, M. Maggi, S. My, S. Nuzzo, A. Pompili, G. Pugliese, R. Radogna, G. Selvaggi, A. Sharma, L. Silvestris, R. Venditti, G. Abbiendi, A. C. Benvenuti, D. Bonacorsi, S. Braibant-Giacomelli, L. Brigliadori, R. Campanini, P. Capiluppi, A. Castro, F. R. Cavallo, G. Codispoti, M. Cuffiani, G. M. Dallavalle, F. Fabbri, A. Fanfani, D. Fasanella, P. Giacomelli, C. Grandi, L. Guiducci, S. Marcellini, G. Masetti, A. Montanari, F. L. Navarria, A. Perrotta, F. Primavera, A. M. Rossi, F. Primavera, T. Rovelli, G. P. Siroli, N. Tosi, R. Travaglini, S. Albergo, G. Cappello, M. Chiorboli, S. Costa, F. Giordano, R. Potenza, A. Tricomi, C. Tuve, G. Barbagli, V. Ciulli, C. Civinini, R. D’Alessandro, E. Focardi, E. Gallo, S. Gonzi, V. Gori, P. Lenzi, M. Meschini, S. Paoletti, G. Sguazzoni, A. Tropiano, L. Benussi, S. Bianco, F. Fabbri, D. Piccolo, R. Ferretti, F. Ferro, M. Lo Vetere, E. Robutti, S. Tosi, M. E. Dinardo, S. Fiorendi, S. Gennai, R. Gerosa, A. Ghezzi, P. Govoni, M. T. Lucchini, S. Malvezzi, R. A. Manzoni, A. Martelli, B. Marzocchi, D. Menasce, L. Moroni, M. Paganoni, D. Pedrini, S. Ragazzi, N. Redaelli, T. Tabarelli de Fatis, S. Buontempo, N. Cavallo, S. Di Guida, F. Fabozzi, A. O. M. Iorio, L. Lista, S. Meola, M. Merola, P. Paolucci, P. Azzi, N. Bacchetta, D. Bisello, A. Branca, R. Carlin, P. Checchia, M. Dall’Osso, T. Dorigo, M. Galanti, U. Gasparini, P. Giubilato, A. Gozzelino, S. Lacaprara, M. Margoni, A. T. Meneguzzo, M. Passaseo, J. Pazzini, M. Pegoraro, N. Pozzobon, P. Ronchese, F. Simonetto, E. Torassa, M. Tosi, A. Triossi, P. Zotto, A. Zucchetta, G. Zumerle, M. Gabusi, S. P. Ratti, V. Re, C. Riccardi, P. Salvini, P. Vitulo, M. Biasini, G. M. Bilei, D. Ciangottini, L. Fanò, P. Lariccia, G. Mantovani, M. Menichelli, A. Saha, A. Santocchia, A. Spiezia, K. Androsov, P. Azzurri, G. Bagliesi, J. Bernardini, T. Boccali, G. Broccolo, R. Castaldi, M. A. Ciocci, R. Dell’Orso, S. Donato, F. Fiori, L. Foà, A. Giassi, M. T. Grippo, F. Ligabue, T. Lomtadze, L. Martini, A. Messineo, C. S. Moon, F. Palla, A. Rizzi, A. Savoy-Navarro, A. T. Serban, P. Spagnolo, P. Squillacioti, R. Tenchini, G. Tonelli, A. Venturi, P. G. Verdini, C. Vernieri, L. Barone, F. Cavallari, G. D’imperio, D. Del Re, M. Diemoz, C. Jorda, E. Longo, F. Margaroli, P. Meridiani, F. Micheli, S. Nourbakhsh, G. Organtini, R. Paramatti, S. Rahatlou, C. Rovelli, F. Santanastasio, L. Soffi, P. Traczyk, N. Amapane, R. Arcidiacono, S. Argiro, M. Arneodo, R. Bellan, C. Biino, N. Cartiglia, S. Casasso, M. Costa, A. Degano, N. Demaria, L. Finco, C. Mariotti, S. Maselli, G. Mazza, E. Migliore, V. Monaco, M. Musich, M. M. Obertino, G. Ortona, L. Pacher, N. Pastrone, M. Pelliccioni, G. L. Pinna Angioni, A. Potenza, A. Romero, M. Ruspa, R. Sacchi, A. Solano, A. Staiano, S. Belforte, V. Candelise, M. Casarsa, F. Cossutti, G. Della Ricca, B. Gobbo, C. La Licata, M. Marone, A. Schizzi, T. Umer, A. Zanetti, S. Chang, T. A. Kropivnitskaya, S. K. Nam, D. H. Kim, G. N. Kim, M. S. Kim, M. S. Kim, D. J. Kong, S. Lee, Y. D. Oh, H. Park, A. Sakharov, D. C. Son, T. J. Kim, J. Y. Kim, S. Song, S. Choi, D. Gyun, B. Hong, M. Jo, H. Kim, Y. Kim, B. Lee, K. S. Lee, S. K. Park, Y. Roh, M. Choi, J. H. Kim, I. C. Park, G. Ryu, M. S. Ryu, Y. Choi, Y. K. Choi, J. Goh, D. Kim, E. Kwon, J. Lee, H. Seo, I. Yu, A. Juodagalvis, J. R. Komaragiri, M. A. B. Md Ali, E. Casimiro Linares, H. Castilla-Valdez, E. De La Cruz-Burelo, I. Heredia-de La Cruz, A. Hernandez-Almada, R. Lopez-Fernandez, A. Sanchez-Hernandez, S. Carrillo Moreno, F. Vazquez Valencia, I. Pedraza, H. A. Salazar Ibarguen, A. Morelos Pineda, D. Krofcheck, P. H. Butler, S. Reucroft, A. Ahmad, M. Ahmad, Q. Hassan, H. R. Hoorani, W. A. Khan, T. Khurshid, M. Shoaib, H. Bialkowska, M. Bluj, B. Boimska, T. Frueboes, M. Górski, M. Kazana, K. Nawrocki, K. Romanowska-Rybinska, M. Szleper, P. Zalewski, G. Brona, K. Bunkowski, M. Cwiok, W. Dominik, K. Doroba, A. Kalinowski, M. Konecki, J. Krolikowski, M. Misiura, M. Olszewski, W. Wolszczak, P. Bargassa, C. Beir ao Da Cruz E Silva, P. Faccioli, P. G. Ferreira Parracho, M. Gallinaro, L. Lloret Iglesias, F. Nguyen, J. Rodrigues Antunes, J. Seixas, J. Varela, P. Vischia, P. Bunin, M. Gavrilenko, I. Golutvin, A. Kamenev, V. Karjavin, V. Konoplyanikov, V. Korenkov, A. Lanev, A. Malakhov, V. Matveev, V. V. Mitsyn, P. Moisenz, V. Palichik, V. Perelygin, S. Shmatov, V. Smirnov, E. Tikhonenko, A. Zarubin, V. Golovtsov, Y. Ivanov, V. Kim, P. Levchenko, V. Murzin, V. Oreshkin, I. Smirnov, V. Sulimov, L. Uvarov, S. Vavilov, A. Vorobyev, An. Vorobyev, Yu. Andreev, A. Dermenev, S. Gninenko, N. Golubev, M. Kirsanov, N. Krasnikov, A. Pashenkov, D. Tlisov, A. Toropin, V. Epshteyn, V. Gavrilov, N. Lychkovskaya, V. Popov, I. Pozdnyakov, G. Safronov, S. Semenov, A. Spiridonov, V. Stolin, E. Vlasov, A. Zhokin, V. Andreev, M. Azarkin, I. Dremin, M. Kirakosyan, A. Leonidov, G. Mesyats, S. V. Rusakov, A. Vinogradov, A. Belyaev, E. Boos, V. Bunichev, M. Dubinin, L. Dudko, A. Ershov, A. Gribushin, V. Klyukhin, I. Lokhtin, S. Obraztsov, M. Perfilov, S. Petrushanko, V. Savrin, I. Azhgirey, I. Bayshev, S. Bitioukov, V. Kachanov, A. Kalinin, D. Konstantinov, V. Krychkine, V. Petrov, R. Ryutin, A. Sobol, L. Tourtchanovitch, S. Troshin, N. Tyurin, A. Uzunian, A. Volkov, P. Adzic, M. Ekmedzic, J. Milosevic, V. Rekovic, J. Alcaraz Maestre, C. Battilana, E. Calvo, M. Cerrada, M. Chamizo Llatas, N. Colino, B. De La Cruz, A. Delgado Peris, D. Domínguez Vázquez, A. Escalante Del Valle, C. Fernandez Bedoya, J. P. Fernández Ramos, J. Flix, M. C. Fouz, P. Garcia-Abia, O. Gonzalez Lopez, S. Goy Lopez, J. M. Hernandez, M. I. Josa, E. Navarro De Martino, A. Pérez-Calero Yzquierdo, J. Puerta Pelayo, A. Quintario Olmeda, I. Redondo, L. Romero, M. S. Soares, C. Albajar, J. F. de Trocóniz, M. Missiroli, D. Moran, H. Brun, J. Cuevas, J. Fernandez Menendez, S. Folgueras, I. Gonzalez Caballero, J. A. Brochero Cifuentes, I. J. Cabrillo, A. Calderon, J. Duarte Campderros, M. Fernandez, G. Gomez, A. Graziano, A. Lopez Virto, J. Marco, R. Marco, C. Martinez Rivero, F. Matorras, F. J. Munoz Sanchez, J. Piedra Gomez, T. Rodrigo, A. Y. Rodríguez-Marrero, A. Ruiz-Jimeno, L. Scodellaro, I. Vila, R. Vilar Cortabitarte, D. Abbaneo, E. Auffray, G. Auzinger, M. Bachtis, P. Baillon, A. H. Ball, D. Barney, A. Benaglia, J. Bendavid, L. Benhabib, J. F. Benitez, C. Bernet, G. Bianchi, P. Bloch, A. Bocci, A. Bonato, O. Bondu, C. Botta, H. Breuker, T. Camporesi, G. Cerminara, S. Colafranceschi, M. D’Alfonso, D. d’Enterria, A. Dabrowski, A. David, F. De Guio, A. De Roeck, S. De Visscher, E. Di Marco, M. Dobson, M. Dordevic, B. Dorney, N. Dupont-Sagorin, A. Elliott-Peisert, J. Eugster, G. Franzoni, W. Funk, D. Gigi, K. Gill, D. Giordano, M. Girone, F. Glege, R. Guida, S. Gundacker, M. Guthoff, J. Hammer, M. Hansen, P. Harris, J. Hegeman, V. Innocente, P. Janot, K. Kousouris, K. Krajczar, P. Lecoq, C. Lourenço, N. Magini, L. Malgeri, M. Mannelli, J. Marrouche, L. Masetti, F. Meijers, S. Mersi, E. Meschi, F. Moortgat, S. Morovic, M. Mulders, P. Musella, L. Orsini, L. Pape, E. Perez, L. Perrozzi, A. Petrilli, G. Petrucciani, A. Pfeiffer, M. Pierini, M. Pimiä, D. Piparo, M. Plagge, A. Racz, G. Rolandi, M. Rovere, H. Sakulin, C. Schäfer, C. Schwick, A. Sharma, P. Siegrist, P. Silva, M. Simon, P. Sphicas, D. Spiga, J. Steggemann, B. Stieger, M. Stoye, Y. Takahashi, D. Treille, A. Tsirou, G. I. Veres, N. Wardle, H. K. Wöhri, H. Wollny, W. D. Zeuner, W. Bertl, K. Deiters, W. Erdmann, R. Horisberger, Q. Ingram, H. C. Kaestli, D. Kotlinski, U. Langenegger, D. Renker, T. Rohe, F. Bachmair, L. Bäni, L. Bianchini, M. A. Buchmann, B. Casal, N. Chanon, G. Dissertori, M. Dittmar, M. Donegà, M. Dünser, P. Eller, C. Grab, D. Hits, J. Hoss, W. Lustermann, B. Mangano, A. C. Marini, P. Martinez Ruiz del Arbol, M. Masciovecchio, D. Meister, N. Mohr, C. Nägeli, F. Nessi-Tedaldi, F. Pandolfi, F. Pauss, M. Peruzzi, M. Quittnat, L. Rebane, M. Rossini, A. Starodumov, M. Takahashi, K. Theofilatos, R. Wallny, H. A. Weber, C. Amsler, M. F. Canelli, V. Chiochia, A. De Cosa, A. Hinzmann, T. Hreus, B. Kilminster, C. Lange, B. Millan Mejias, J. Ngadiuba, P. Robmann, F. J. Ronga, S. Taroni, M. Verzetti, Y. Yang, M. Cardaci, K. H. Chen, C. Ferro, C. M. Kuo, W. Lin, Y. J. Lu, R. Volpe, S. S. Yu, P. Chang, Y. H. Chang, Y. W. Chang, Y. Chao, K. F. Chen, P. H. Chen, C. Dietz, U. Grundler, W.-S. Hou, K. Y. Kao, Y. J. Lei, Y. F. Liu, R.-S. Lu, D. Majumder, E. Petrakou, Y. M. Tzeng, R. Wilken, B. Asavapibhop, G. Singh, N. Srimanobhas, N. Suwonjandee, A. Adiguzel, M. N. Bakirci, S. Cerci, C. Dozen, I. Dumanoglu, E. Eskut, S. Girgis, G. Gokbulut, E. Gurpinar, I. Hos, E. E. Kangal, A. Kayis Topaksu, G. Onengut, K. Ozdemir, S. Ozturk, A. Polatoz, D. Sunar Cerci, B. Tali, H. Topakli, M. Vergili, C. Zorbilmez, I. V. Akin, B. Bilin, S. Bilmis, H. Gamsizkan, B. Isildak, G. Karapinar, K. Ocalan, S. Sekmen, U. E. Surat, M. Yalvac, M. Zeyrek, A. Albayrak, E. Gülmez, M. Kaya, O. Kaya, T. Yetkin, K. Cankocak, F. I. Vardarlı, L. Levchuk, P. Sorokin, J. J. Brooke, E. Clement, D. Cussans, H. Flacher, J. Goldstein, M. Grimes, G. P. Heath, H. F. Heath, J. Jacob, L. Kreczko, C. Lucas, Z. Meng, D. M. Newbold, S. Paramesvaran, A. Poll, T. Sakuma, S. Senkin, V. J. Smith, T. Williams, K. W. Bell, A. Belyaev, C. Brew, R. M. Brown, D. J. A. Cockerill, J. A. Coughlan, K. Harder, S. Harper, E. Olaiya, D. Petyt, C. H. Shepherd-Themistocleous, A. Thea, I. R. Tomalin, W. J. Womersley, S. D. Worm, M. Baber, R. Bainbridge, O. Buchmuller, D. Burton, D. Colling, N. Cripps, M. Cutajar, P. Dauncey, G. Davies, M. Della Negra, P. Dunne, W. Ferguson, J. Fulcher, D. Futyan, G. Hall, G. Iles, M. Jarvis, G. Karapostoli, M. Kenzie, R. Lane, R. Lucas, L. Lyons, A.-M. Magnan, S. Malik, B. Mathias, J. Nash, A. Nikitenko, J. Pela, M. Pesaresi, K. Petridis, D. M. Raymond, S. Rogerson, A. Rose, C. Seez, P. Sharp, A. Tapper, M. Vazquez Acosta, T. Virdee, S. C. Zenz, J. E. Cole, P. R. Hobson, A. Khan, P. Kyberd, D. Leggat, D. Leslie, W. Martin, I. D. Reid, P. Symonds, L. Teodorescu, M. Turner, J. Dittmann, K. Hatakeyama, A. Kasmi, H. Liu, T. Scarborough, O. Charaf, S. I. Cooper, C. Henderson, P. Rumerio, A. Avetisyan, T. Bose, C. Fantasia, P. Lawson, C. Richardson, J. Rohlf, J. St. John, L. Sulak, J. Alimena, E. Berry, S. Bhattacharya, G. Christopher, D. Cutts, Z. Demiragli, N. Dhingra, A. Ferapontov, A. Garabedian, U. Heintz, G. Kukartsev, E. Laird, G. Landsberg, M. Luk, M. Narain, S. Segala, T. Sinthuprasith, T. Speer, J. Swanson, R. Breedon, G. Breto, M. Calderon De La Barca Sanchez, S. Chauhan, M. Chertok, J. Conway, R. Conway, P. T. Cox, R. Erbacher, M. Gardner, W. Ko, R. Lander, T. Miceli, M. Mulhearn, D. Pellett, J. Pilot, F. Ricci-Tam, M. Searle, S. Shalhout, J. Smith, M. Squires, D. Stolp, M. Tripathi, S. Wilbur, R. Yohay, R. Cousins, P. Everaerts, C. Farrell, J. Hauser, M. Ignatenko, G. Rakness, E. Takasugi, V. Valuev, M. Weber, K. Burt, R. Clare, J. Ellison, J. W. Gary, G. Hanson, J. Heilman, M. Ivova Rikova, P. Jandir, E. Kennedy, F. Lacroix, O. R. Long, A. Luthra, M. Malberti, M. Olmedo Negrete, A. Shrinivas, S. Sumowidagdo, S. Wimpenny, J. G. Branson, G. B. Cerati, S. Cittolin, R. T. D’Agnolo, A. Holzner, R. Kelley, D. Klein, J. Letts, I. Macneill, D. Olivito, S. Padhi, C. Palmer, M. Pieri, M. Sani, V. Sharma, S. Simon, E. Sudano, M. Tadel, Y. Tu, A. Vartak, C. Welke, F. Würthwein, A. Yagil, D. Barge, J. Bradmiller-Feld, C. Campagnari, T. Danielson, A. Dishaw, V. Dutta, K. Flowers, M. Franco Sevilla, P. Geffert, C. George, F. Golf, L. Gouskos, J. Incandela, C. Justus, N. Mccoll, J. Richman, D. Stuart, W. To, C. West, J. Yoo, A. Apresyan, A. Bornheim, J. Bunn, Y. Chen, J. Duarte, A. Mott, H. B. Newman, C. Pena, C. Rogan, M. Spiropulu, V. Timciuc, J. R. Vlimant, R. Wilkinson, S. Xie, R. Y. Zhu, V. Azzolini, A. Calamba, B. Carlson, T. Ferguson, Y. Iiyama, M. Paulini, J. Russ, H. Vogel, I. Vorobiev, J. P. Cumalat, W. T. Ford, A. Gaz, M. Krohn, E. Luiggi Lopez, U. Nauenberg, J. G. Smith, K. Stenson, K. A. Ulmer, S. R. Wagner, J. Alexander, A. Chatterjee, J. Chaves, J. Chu, S. Dittmer, N. Eggert, N. Mirman, G. Nicolas Kaufman, J. R. Patterson, A. Ryd, E. Salvati, L. Skinnari, W. Sun, W. D. Teo, J. Thom, J. Thompson, J. Tucker, Y. Weng, L. Winstrom, P. Wittich, D. Winn, S. Abdullin, M. Albrow, J. Anderson, G. Apollinari, L. A. T. Bauerdick, A. Beretvas, J. Berryhill, P. C. Bhat, G. Bolla, K. Burkett, J. N. Butler, H. W. K. Cheung, F. Chlebana, S. Cihangir, V. D. Elvira, I. Fisk, J. Freeman, Y. Gao, E. Gottschalk, L. Gray, D. Green, S. Grünendahl, O. Gutsche, J. Hanlon, D. Hare, R. M. Harris, J. Hirschauer, B. Hooberman, S. Jindariani, M. Johnson, U. Joshi, K. Kaadze, B. Klima, B. Kreis, S. Kwan, J. Linacre, D. Lincoln, R. Lipton, T. Liu, J. Lykken, K. Maeshima, J. M. Marraffino, V. I. Martinez Outschoorn, S. Maruyama, D. Mason, P. McBride, P. Merkel, K. Mishra, S. Mrenna, Y. Musienko, S. Nahn, C. Newman-Holmes, V. O’Dell, O. Prokofyev, E. Sexton-Kennedy, S. Sharma, A. Soha, W. J. Spalding, L. Spiegel, L. Taylor, S. Tkaczyk, N. V. Tran, L. Uplegger, E. W. Vaandering, R. Vidal, A. Whitbeck, J. Whitmore, F. Yang, D. Acosta, P. Avery, P. Bortignon, D. Bourilkov, M. Carver, T. Cheng, D. Curry, S. Das, M. De Gruttola, G. P. Di Giovanni, R. D. Field, M. Fisher, I. K. Furic, J. Hugon, J. Konigsberg, A. Korytov, T. Kypreos, J. F. Low, K. Matchev, P. Milenovic, G. Mitselmakher, L. Muniz, A. Rinkevicius, L. Shchutska, M. Snowball, D. Sperka, J. Yelton, M. Zakaria, S. Hewamanage, S. Linn, P. Markowitz, G. Martinez, J. L. Rodriguez, T. Adams, A. Askew, J. Bochenek, B. Diamond, J. Haas, S. Hagopian, V. Hagopian, K. F. Johnson, H. Prosper, V. Veeraraghavan, M. Weinberg, M. M. Baarmand, M. Hohlmann, H. Kalakhety, F. Yumiceva, M. R. Adams, L. Apanasevich, V. E. Bazterra, D. Berry, R. R. Betts, I. Bucinskaite, R. Cavanaugh, O. Evdokimov, L. Gauthier, C. E. Gerber, D. J. Hofman, S. Khalatyan, P. Kurt, D. H. Moon, C. O’Brien, C. Silkworth, P. Turner, N. Varelas, B. Bilki, W. Clarida, K. Dilsiz, F. Duru, M. Haytmyradov, J.-P. Merlo, H. Mermerkaya, A. Mestvirishvili, A. Moeller, J. Nachtman, H. Ogul, Y. Onel, F. Ozok, A. Penzo, R. Rahmat, S. Sen, P. Tan, E. Tiras, J. Wetzel, K. Yi, B. A. Barnett, B. Blumenfeld, S. Bolognesi, D. Fehling, A. V. Gritsan, P. Maksimovic, C. Martin, M. Swartz, P. Baringer, A. Bean, G. Benelli, C. Bruner, R. P. Kenny, M. Malek, M. Murray, D. Noonan, S. Sanders, J. Sekaric, R. Stringer, Q. Wang, J. S. Wood, I. Chakaberia, A. Ivanov, S. Khalil, M. Makouski, Y. Maravin, L. K. Saini, S. Shrestha, N. Skhirtladze, I. Svintradze, J. Gronberg, D. Lange, F. Rebassoo, D. Wright, A. Baden, A. Belloni, B. Calvert, S. C. Eno, J. A. Gomez, N. J. Hadley, R. G. Kellogg, T. Kolberg, Y. Lu, M. Marionneau, A. C. Mignerey, K. Pedro, A. Skuja, M. B. Tonjes, S. C. Tonwar, A. Apyan, R. Barbieri, G. Bauer, W. Busza, I. A. Cali, M. Chan, L. Di Matteo, G. Gomez Ceballos, M. Goncharov, D. Gulhan, M. Klute, Y. S. Lai, Y.-J. Lee, A. Levin, P. D. Luckey, T. Ma, C. Paus, D. Ralph, C. Roland, G. Roland, G. S. F. Stephans, F. Stöckli, K. Sumorok, D. Velicanu, J. Veverka, B. Wyslouch, M. Yang, M. Zanetti, V. Zhukova, B. Dahmes, A. Gude, S. C. Kao, K. Klapoetke, Y. Kubota, J. Mans, N. Pastika, R. Rusack, A. Singovsky, N. Tambe, J. Turkewitz, J. G. Acosta, S. Oliveros, E. Avdeeva, K. Bloom, S. Bose, D. R. Claes, A. Dominguez, R. Gonzalez Suarez, J. Keller, D. Knowlton, I. Kravchenko, J. Lazo-Flores, S. Malik, F. Meier, G. R. Snow, M. Zvada, J. Dolen, A. Godshalk, I. Iashvili, A. Kharchilava, A. Kumar, S. Rappoccio, G. Alverson, E. Barberis, D. Baumgartel, M. Chasco, J. Haley, A. Massironi, D. M. Morse, D. Nash, T. Orimoto, D. Trocino, R. -J. Wang, D. Wood, J. Zhang, K. A. Hahn, A. Kubik, N. Mucia, N. Odell, B. Pollack, A. Pozdnyakov, M. Schmitt, S. Stoynev, K. Sung, M. Velasco, S. Won, A. Brinkerhoff, K. M. Chan, A. Drozdetskiy, M. Hildreth, C. Jessop, D. J. Karmgard, N. Kellams, K. Lannon, W. Luo, S. Lynch, N. Marinelli, T. Pearson, M. Planer, R. Ruchti, N. Valls, M. Wayne, M. Wolf, A. Woodard, L. Antonelli, J. Brinson, B. Bylsma, L. S. Durkin, S. Flowers, A. Hart, C. Hill, R. Hughes, K. Kotov, T. Y. Ling, D. Puigh, M. Rodenburg, G. Smith, B. L. Winer, H. Wolfe, H. W. Wulsin, O. Driga, P. Elmer, J. Hardenbrook, P. Hebda, A. Hunt, S. A. Koay, P. Lujan, D. Marlow, T. Medvedeva, M. Mooney, J. Olsen, P. Piroué, X. Quan, H. Saka, D. Stickland, C. Tully, J. S. Werner, A. Zuranski, E. Brownson, H. Mendez, J. E. Ramirez Vargas, V. E. Barnes, D. Benedetti, D. Bortoletto, M. De Mattia, L. Gutay, Z. Hu, M. K. Jha, M. Jones, K. Jung, M. Kress, N. Leonardo, D. Lopes Pegna, V. Maroussov, D. H. Miller, N. Neumeister, B. C. Radburn-Smith, X. Shi, I. Shipsey, D. Silvers, A. Svyatkovskiy, F. Wang, W. Xie, L. Xu, H. D. Yoo, J. Zablocki, Y. Zheng, N. Parashar, J. Stupak, A. Adair, B. Akgun, K. M. Ecklund, F. J. M. Geurts, W. Li, B. Michlin, B. P. Padley, R. Redjimi, J. Roberts, J. Zabel, B. Betchart, A. Bodek, R. Covarelli, P. de Barbaro, R. Demina, Y. Eshaq, T. Ferbel, A. Garcia-Bellido, P. Goldenzweig, J. Han, A. Harel, A. Khukhunaishvili, S. Korjenevski, G. Petrillo, D. Vishnevskiy, R. Ciesielski, L. Demortier, K. Goulianos, G. Lungu, C. Mesropian, S. Arora, A. Barker, J. P. Chou, C. Contreras-Campana, E. Contreras-Campana, D. Duggan, D. Ferencek, Y. Gershtein, R. Gray, E. Halkiadakis, D. Hidas, S. Kaplan, A. Lath, S. Panwalkar, M. Park, R. Patel, S. Salur, S. Schnetzer, S. Somalwar, R. Stone, S. Thomas, P. Thomassen, M. Walker, K. Rose, S. Spanier, A. York, O. Bouhali, A. Castaneda Hernandez, R. Eusebi, W. Flanagan, J. Gilmore, T. Kamon, V. Khotilovich, V. Krutelyov, R. Montalvo, I. Osipenkov, Y. Pakhotin, A. Perloff, J. Roe, A. Rose, A. Safonov, I. Suarez, A. Tatarinov, N. Akchurin, C. Cowden, J. Damgov, C. Dragoiu, P. R. Dudero, J. Faulkner, K. Kovitanggoon, S. Kunori, S. W. Lee, T. Libeiro, I. Volobouev, E. Appelt, A. G. Delannoy, S. Greene, A. Gurrola, W. Johns, C. Maguire, Y. Mao, A. Melo, M. Sharma, P. Sheldon, B. Snook, S. Tuo, J. Velkovska, M. W. Arenton, S. Boutle, B. Cox, B. Francis, J. Goodell, R. Hirosky, A. Ledovskoy, H. Li, C. Lin, C. Neu, J. Wood, C. Clarke, R. Harr, P. E. Karchin, C. Kottachchi Kankanamge Don, P. Lamichhane, J. Sturdy, D. A. Belknap, D. Carlsmith, M. Cepeda, S. Dasu, L. Dodd, S. Duric, E. Friis, R. Hall-Wilton, M. Herndon, A. Hervé, P. Klabbers, A. Lanaro, C. Lazaridis, A. Levine, R. Loveless, A. Mohapatra, I. Ojalvo, T. Perry, G. A. Pierro, G. Polese, I. Ross, T. Sarangi, A. Savin, W. H. Smith, D. Taylor, P. Verwilligen, C. Vuosalo, N. Woods, [Authorinst]The CMS Collaboration

**Affiliations:** Yerevan Physics Institute, Yerevan, Armenia; Institut für Hochenergiephysik der OeAW, Vienna, Austria; National Centre for Particle and High Energy Physics, Minsk, Belarus; Universiteit Antwerpen, Antwerp, Belgium; Vrije Universiteit Brussel, Brussels, Belgium; Université Libre de Bruxelles, Brussels, Belgium; Ghent University, Ghent, Belgium; Université Catholique de Louvain, Louvain-la-Neuve, Belgium; Université de Mons, Mons, Belgium; Centro Brasileiro de Pesquisas Fisicas, Rio de Janeiro, Brazil; Universidade do Estado do Rio de Janeiro, Rio de Janeiro, Brazil; Universidade Estadual Paulista, Universidade Federal do ABC, São Paulo, Brazil; Institute for Nuclear Research and Nuclear Energy, Sofia, Bulgaria; University of Sofia, Sofia, Bulgaria; Institute of High Energy Physics, Beijing, China; State Key Laboratory of Nuclear Physics and Technology, Peking University, Beijing, China; Universidad de Los Andes, Bogotá, Colombia; Faculty of Electrical Engineering, Mechanical Engineering and Naval Architecture, University of Split, Split, Croatia; Faculty of Science, University of Split, Split, Croatia; Institute Rudjer Boskovic, Zagreb, Croatia; University of Cyprus, Nicosia, Cyprus; Charles University, Prague, Czech Republic; Academy of Scientific Research and Technology of the Arab Republic of Egypt, Egyptian Network of High Energy Physics, Cairo, Egypt; National Institute of Chemical Physics and Biophysics, Tallinn, Estonia; Department of Physics, University of Helsinki, Helsinki, Finland; Helsinki Institute of Physics, Helsinki, Finland; Lappeenranta University of Technology, Lappeenranta, Finland; DSM/IRFU, CEA/Saclay, Gif-sur-Yvette, France; Laboratoire Leprince-Ringuet, Ecole Polytechnique, IN2P3-CNRS, Palaiseau, France; Institut Pluridisciplinaire Hubert Curien, Université de Strasbourg, Université de Haute Alsace Mulhouse, CNRS/IN2P3, Strasbourg, France; Centre de Calcul de l’Institut National de Physique Nucleaire et de Physique des Particules, CNRS/IN2P3, Villeurbanne, France; Institut de Physique Nucléaire de Lyon, Université de Lyon, Université Claude Bernard Lyon 1, CNRS-IN2P3, Villeurbanne, France; Institute of High Energy Physics and Informatization, Tbilisi State University, Tbilisi, Georgia; I. Physikalisches Institut, RWTH Aachen University, Aachen, Germany; III. Physikalisches Institut A, RWTH Aachen University, Aachen, Germany; III. Physikalisches Institut B, RWTH Aachen University, Aachen, Germany; Deutsches Elektronen-Synchrotron, Hamburg, Germany; University of Hamburg, Hamburg, Germany; Institut für Experimentelle Kernphysik, Karlsruhe, Germany; Institute of Nuclear and Particle Physics (INPP), NCSR Demokritos, Aghia Paraskevi, Greece; University of Athens, Athens, Greece; University of Ioánnina, Ioánnina, Greece; Wigner Research Centre for Physics, Budapest, Hungary; Institute of Nuclear Research ATOMKI, Debrecen, Hungary; University of Debrecen, Debrecen, Hungary; National Institute of Science Education and Research, Bhubaneswar, India; Panjab University, Chandigarh, India; University of Delhi, Delhi, India; Saha Institute of Nuclear Physics, Kolkata, India; Bhabha Atomic Research Centre, Mumbai, India; Tata Institute of Fundamental Research, Mumbai, India; Institute for Research in Fundamental Sciences (IPM), Tehran, Iran; University College Dublin, Dublin, Ireland; INFN Sezione di Bari, Università di Bari, Politecnico di Bari, Bari, Italy; INFN Sezione di Bologna, Università di Bologna, Bologna, Italy; INFN Sezione di Catania, Università di Catania, CSFNSM, Catania, Italy; INFN Sezione di Firenze, Università di Firenze, Florence, Italy; INFN Laboratori Nazionali di Frascati, Frascati, Italy; INFN Sezione di Genova, Università di Genova, Genoa, Italy; INFN Sezione di Milano-Bicocca, Università di Milano-Bicocca, Milan, Italy; INFN Sezione di Napoli, Università di Napoli ‘Federico II’, Naples, Italy, Università della Basilicata, Potenza, Italy, Università G. Marconi, Rome, Italy; INFN Sezione di Padova, Università di Padova, Padua, Italy, Università di Trento, Trento, Italy; INFN Sezione di Pavia, Università di Pavia, Pavia, Italy; INFN Sezione di Perugia, Università di Perugia, Perugia, Italy; INFN Sezione di Pisa, Università di Pisa, Scuola Normale Superiore di Pisa, Pisa, Italy; INFN Sezione di Roma, Università di Roma, Rome, Italy; INFN Sezione di Torino, Università di Torino, Turin, Italy, Università del Piemonte Orientale, Novara, Italy; INFN Sezione di Trieste, Università di Trieste, Trieste, Italy; Kangwon National University, Chunchon, Korea; Kyungpook National University, Daegu, Korea; Chonbuk National University, Jeonju, Korea; Chonnam National University, Institute for Universe and Elementary Particles, Kwangju, Korea; Korea University, Seoul, Korea; University of Seoul, Seoul, Korea; Sungkyunkwan University, Suwon, Korea; Vilnius University, Vilnius, Lithuania; National Centre for Particle Physics, Universiti Malaya, Kuala Lumpur, Malaysia; Centro de Investigacion y de Estudios Avanzados del IPN, Mexico City, Mexico; Universidad Iberoamericana, Mexico City, Mexico; Benemerita Universidad Autonoma de Puebla, Puebla, Mexico; Universidad Autónoma de San Luis Potosí, San Luis Potosí, Mexico; University of Auckland, Auckland, New Zealand; University of Canterbury, Christchurch, New Zealand; National Centre for Physics, Quaid-I-Azam University, Islamabad, Pakistan; National Centre for Nuclear Research, Swierk, Poland; Institute of Experimental Physics, Faculty of Physics, University of Warsaw, Warsaw, Poland; Laboratório de Instrumentação e Física Experimental de Partículas, Lisbon, Portugal; Joint Institute for Nuclear Research, Dubna, Russia; Petersburg Nuclear Physics Institute, Gatchina, (St. Petersburg), Russia; Institute for Nuclear Research, Moscow, Russia; Institute for Theoretical and Experimental Physics, Moscow, Russia; P. N. Lebedev Physical Institute, Moscow, Russia; Skobeltsyn Institute of Nuclear Physics, Lomonosov Moscow State University, Moscow, Russia; State Research Center of Russian Federation, Institute for High Energy Physics, Protvino, Russia; Faculty of Physics and Vinca Institute of Nuclear Sciences, University of Belgrade, Belgrade, Serbia; Centro de Investigaciones Energéticas Medioambientales y Tecnológicas (CIEMAT), Madrid, Spain; Universidad Autónoma de Madrid, Madrid, Spain; Universidad de Oviedo, Oviedo, Spain; Instituto de Física de Cantabria (IFCA), CSIC-Universidad de Cantabria, Santander, Spain; CERN, European Organization for Nuclear Research, Geneva, Switzerland; Paul Scherrer Institut, Villigen, Switzerland; Institute for Particle Physics, ETH Zurich, Zurich, Switzerland; Universität Zürich, Zurich, Switzerland; National Central University, Chung-Li, Taiwan; National Taiwan University (NTU), Taipei, Taiwan; Department of Physics, Faculty of Science, Chulalongkorn University, Bangkok, Thailand; Cukurova University, Adana, Turkey; Physics Department, Middle East Technical University, Ankara, Turkey; Bogazici University, Istanbul, Turkey; Istanbul Technical University, Istanbul, Turkey; National Scientific Center, Kharkov Institute of Physics and Technology, Kharkov, Ukraine; University of Bristol, Bristol, UK; Rutherford Appleton Laboratory, Didcot, UK; Imperial College, London, UK; Brunel University, Uxbridge, UK; Baylor University, Waco, USA; The University of Alabama, Tuscaloosa, USA; Boston University, Boston, USA; Brown University, Providence, USA; University of California, Davis, Davis, USA; University of California, Los Angeles, USA; University of California, Riverside, Riverside, USA; University of California, San Diego, La Jolla, USA; University of California, Santa Barbara, Santa Barbara, USA; California Institute of Technology, Pasadena, USA; Carnegie Mellon University, Pittsburgh, USA; University of Colorado at Boulder, Boulder, USA; Cornell University, Ithaca, USA; Fairfield University, Fairfield, USA; Fermi National Accelerator Laboratory, Batavia, USA; University of Florida, Gainesville, USA; Florida International University, Miami, USA; Florida State University, Tallahassee, USA; Florida Institute of Technology, Melbourne, USA; University of Illinois at Chicago (UIC), Chicago, USA; The University of Iowa, Iowa City, USA; Johns Hopkins University, Baltimore, USA; The University of Kansas, Lawrence, USA; Kansas State University, Manhattan, USA; Lawrence Livermore National Laboratory, Livermore, USA; University of Maryland, College Park, USA; Massachusetts Institute of Technology, Cambridge, USA; University of Minnesota, Minneapolis, USA; University of Mississippi, Oxford, USA; University of Nebraska-Lincoln, Lincoln, USA; State University of New York at Buffalo, Buffalo, USA; Northeastern University, Boston, USA; Northwestern University, Evanston, USA; University of Notre Dame, Notre Dame, USA; The Ohio State University, Columbus, USA; Princeton University, Princeton, USA; University of Puerto Rico, Mayaguez, USA; Purdue University, West Lafayette, USA; Purdue University Calumet, Hammond, USA; Rice University, Houston, USA; University of Rochester, Rochester, USA; The Rockefeller University, New York, USA; Rutgers, The State University of New Jersey, Piscataway, USA; University of Tennessee, Knoxville, USA; Texas A&M University, College Station, USA; Texas Tech University, Lubbock, USA; Vanderbilt University, Nashville, USA; University of Virginia, Charlottesville, USA; Wayne State University, Detroit, USA; University of Wisconsin, Madison, USA; CERN, Geneva, Switzerland

## Abstract

The normalized differential cross section for top quark pair ($${\mathrm{t}}\overline{{\mathrm{t}}}$$) production is measured in pp collisions at a centre-of-mass energy of 8$$\,\text {TeV}$$ at the CERN LHC using the CMS detector in data corresponding to an integrated luminosity of 19.7$$\,\text {fb}^{-1}$$. The measurements are performed in the lepton$$+$$jets ($$\mathrm {e}/\mu $$$$+$$jets) and in the dilepton ($$\mathrm {e}^+\mathrm {e}^-$$, $$\mu ^+ \mu ^- $$, and $$\mathrm {e}^\pm \mu ^{\mp }$$) decay channels. The $${\mathrm{t}}\overline{{\mathrm{t}}}$$ cross section is measured as a function of the kinematic properties of the charged leptons, the jets associated to b quarks, the top quarks, and the $${\mathrm{t}}\overline{{\mathrm{t}}}$$ system. The data are compared with several predictions from perturbative quantum chromodynamic up to approximate next-to-next-to-leading-order precision. No significant deviations are observed relative to the standard model predictions.

**Electronic supplementary material:**

The online version of this article (doi:10.1140/epjc/s10052-015-3709-x) contains supplementary material, which is available to authorized users.

## Introduction

Understanding the production and properties of top quarks is fundamental for testing the quality of the standard model (SM) and for searching for new physical phenomena beyond its scope. The large top quark data samples produced in proton–proton (pp) collisions at the CERN LHC provide access to precision measurements that are crucial for checking the internal consistency of the SM at the LHC energy scale. In particular, measurements of the top quark pair (${\mathrm{t}}\overline{{\mathrm{t}}}$) production cross section as a function of ${\mathrm{t}}\overline{{\mathrm{t}}}$ kinematic observables are important for comparing with the state-of-the-art quantum chromodynamic (QCD) predictions within the SM, and thereby constrain QCD parameters. In addition, the top quark plays a relevant role in theories beyond the SM, and such differential measurements are therefore expected to be sensitive to new phenomena [1].

Differential $${\mathrm{t}}\overline{{\mathrm{t}}}$$ production cross sections have been measured previously at the Fermilab $$\mathrm {p}\mathrm {\overline{p}}$$ Tevatron [2, 3], and at the LHC at a centre-of-mass energy $$\sqrt{s}=7$$$$\,\text {TeV}$$  [4–6]. We present here the first measurement of the normalized differential $${\mathrm{t}}\overline{{\mathrm{t}}}$$ production cross section with the CMS detector at $$\sqrt{s}=8$$$$\,\text {TeV}$$. The analysis uses data recorded in 2012 corresponding to an integrated luminosity of $$19.7 \pm 0.5{\,\text {fb}^{-1}} $$, which is about a factor of four larger than the sample used in the measurement performed by the CMS Collaboration at 7$$\,\text {TeV}$$  [5]. The analysis largely follows the procedures of Ref. [5] and benefits from the increase in statistical precision together with improvements in kinematic reconstruction algorithms and extended systematic studies, leading to a significant reduction of the total uncertainties.

The measurements are performed in $\ell +$jets channels ($\ell = \mathrm {e}\text { or }\mu$), which contain a single isolated charged lepton and at least four jets in the final state, and in dilepton channels, with two oppositely charged leptons ($\mathrm {e}^+\mathrm {e}^-$, $\mu ^+ \mu ^-$, $\mathrm {e}^\pm \mu ^{\mp }$) and at least two jets. The ${\mathrm{t}}\overline{{\mathrm{t}}}$ cross section is determined as a function of the kinematic properties of the top quarks and of the ${\mathrm{t}}\overline{{\mathrm{t}}}$ system, as well as of the leptons and jets associated with bottom (b) quarks (b jets) from top quark decays.

The kinematic properties of top quarks are obtained through kinematic-fitting and reconstruction algorithms. The normalized differential ${\mathrm{t}}\overline{{\mathrm{t}}}$ cross section is determined by counting the number of ${\mathrm{t}}\overline{{\mathrm{t}}}$ signal events in each bin of a given observable, correcting for detector effects and acceptance, and dividing by the measured total inclusive ${\mathrm{t}}\overline{{\mathrm{t}}}$ event rate. The latter is evaluated by integrating over all bins in each observable.

The results for directly measured quantities, such as kinematic properties of leptons and b jets, are presented in a fiducial phase space defined by the kinematic and geometric acceptance of all selected final-state objects. This avoids extrapolating the measured cross section into regions that are not experimentally accessible. In addition, the top quark and ${\mathrm{t}}\overline{{\mathrm{t}}}$ distributions are determined in the full phase space, in order to facilitate the comparison with higher-order perturbative QCD calculations. The results are compared to several predictions obtained with the leading-order (LO) MadGraph  [7] generator interfaced to pythia  [8] for parton evolution and hadronization, the next-to-leading-order (NLO) generators powheg  [9–11], interfaced to both pythia and herwig  [12], and mc@nlo [13] interfaced to herwig, and the latest NLO calculations with next-to-next-to-leading-logarithm (NNLL) corrections [14, 15], and approximate next-to-next-to-leading-order (NNLO) predictions [16]. The approximate NNLO predictions can be computed with the DiffTop [17] program.

This document is structured as follows. A brief description of the CMS detector is provided in Sect. 2. Details of the event simulation are given in Sect. 3, and event reconstruction and selection are discussed in Sect. 4. The estimated systematic uncertainties on the measurements of the cross section are described in Sect. 5. The results of the measurement are discussed in Sect. 6, followed by a summary in Sect. 7.

## CMS detector

The central feature of the CMS apparatus is a superconducting solenoid of 13$\text {\,m}$ length and 6$\text {\,m}$ inner diameter, which provides an axial magnetic field of 3.8$\text {\,T}$. Within the field volume are a silicon-pixel and strip tracker, a lead tungstate crystal electromagnetic calorimeter (ECAL), and a brass and scintillator hadron calorimeter (HCAL), each composed of a barrel and two endcap sections. Charged particle trajectories are measured by the inner tracking system, covering a pseudorapidity range of $|\eta |<2.5$. The ECAL and the HCAL surround the tracking volume, providing high-resolution energy and direction measurements of electrons, photons, and hadronic jets up to $|\eta |<3$. Muons are measured in gas-ionization detectors embedded in the steel flux return yoke outside the solenoid covering the region $|\eta |<2.4$. Extensive forward calorimetry complements the coverage provided by the barrel and endcap detectors up to $|\eta |<5.2$. The detector is nearly hermetic, allowing for energy balance measurements in the plane transverse to the beam directions. A two-tier trigger system selects the pp collisions for use in the analysis. A more detailed description of the CMS detector, together with a definition of the coordinate system and the relevant kinematic variables, can be found in Ref. [18].

## Event simulation and theoretical calculations

Event generators, interfaced with a detailed detector simulation, are used to model experimental effects, such as consequences of event reconstruction and choice of selection criteria, as well as detector resolution. The ${\mathrm{t}}\overline{{\mathrm{t}}}$ sample is simulated using the LO MadGraph event generator (v. 5.1.5.11), which implements the relevant matrix elements with up to three additional partons. The MadSpin  [19] package is used to incorporate spin correlation effects with matrix elements for up to three additional partons. The value of the top quark mass is fixed to $m_{{\mathrm{t}}}=172.5\,\text {GeV}$ and the proton structure is described by the parton distribution functions (PDF) CTEQ6L1 [20]. The generated events are subsequently processed with pythia (v. 6.426, referred to as pythia 6 in the following) for parton showering and hadronization, and the MLM prescription [21] is used for matching of matrix-element jets to parton showers. The CMS detector response is simulated using Geant4 (v. 9.4) [22].

In addition to the MadGraph prediction, calculations obtained with the NLO generators mc@nlo (v. 3.41) and powheg (v. 1.0 r1380) are compared to the results presented in Sect. 6. While powheg and mc@nlo are formally equivalent up to the NLO accuracy, they differ in the techniques used to avoid double counting of radiative corrections that can arise from interfacing with the parton showering generators. Two powheg samples are used: one is processed through pythia 6 and the other through herwig (v. 6.520, referred to as herwig 6 in the following) for the subsequent parton showering and hadronization. The parton showering in pythia 6 is based on a transverse-momentum-ordered evolution scale, whereas in herwig 6 it is angular-ordered. The events generated with mc@nlo are interfaced with herwig 6. The herwig 6 AUET2 tune [23] is used to model the underlying event in the powheg$+$herwig 6 sample, while the default tune is used in the mc@nlo$+$herwig 6 sample. The proton structure is described by the PDF sets CT10 [24] and CTEQ6M [20] for powheg and mc@nlo, respectively. In addition, the latest available NLO$+$NNLL [14, 15] and approximate NNLO QCD predictions [16] are also used to compare with the data. The NNLO MSTW2008 [25] PDF set is used for both the NLO$+$NNLL and the approximate NNLO calculations.

Standard model background samples are simulated with MadGraph (without the MadSpin package), powheg, or pythia 6, depending on the process. The main background contributions originate from the production of W and Z/$\gamma ^{*}$ bosons with additional jets (referred to as W$+$jets and Z$+$jets, respectively, in the following), single top quark (*s*-, *t*-, and tW channels), diboson (WW, WZ, and ZZ), ${\mathrm{t}}\overline{{\mathrm{t}}}$ production in association with a Z, W, or $\gamma$ boson (referred to as ${\mathrm{t}}\overline{{\mathrm{t}}}+\text{Z/W/}{\gamma}$ in the following), and QCD multijet events. The W$+$jets, Z$+$jets, and ${\mathrm{t}}\overline{{\mathrm{t}}}+\text{Z/W/}{\gamma}$ samples are simulated with MadGraph with up to two additional partons in the final state. The powheg generator is used for simulating single top quark production, while pythia 6 is used to simulate diboson and QCD multijet events. Parton showering and hadronization are also simulated with pythia 6 in all the background samples. The pythia 6 Z2* tune [26] is used to characterize the underlying event in both the ${\mathrm{t}}\overline{{\mathrm{t}}}$ and the background samples.

For comparison with the measured distributions, the event yields in the simulated samples are normalized to an integrated luminosity of 19.7$\,\text {fb}^{-1}$, according to their predicted cross sections. These are taken from NNLO (W$+$jets [27, 28] and Z$+$jets [27]), NLO$+$NNLL (single top quark *s*-, *t*-, and tW channels [16]), NLO (diboson [29], ${\mathrm{t}}\overline{{\mathrm{t}}}+\text{W}$ [30], and ${\mathrm{t}}\overline{{\mathrm{t}}}+\text{Z}$ [31]), and LO (QCD multijet [8]) calculations. The predicted cross section for the ${\mathrm{t}}\overline{{\mathrm{t}}}+{\gamma}$ sample is obtained by scaling the LO cross section obtained with the Whizard event generator [32] by an NLO/LO correction *K*-factor [33]. Correction factors described in Sects. 4 and 5, and subsequently referred to as scale factors, are applied when needed to improve the description of the data by the simulation. The ${\mathrm{t}}\overline{{\mathrm{t}}}$ simulation is normalized to the data to present the expected rates in the figures in Sect. 4.

## Event reconstruction and selection

The event selection is similar to that described in Ref. [5] for the measurement of normalized differential $${\mathrm{t}}\overline{{\mathrm{t}}}$$ cross sections at $$\sqrt{s} = 7$$$$\,\text {TeV}$$, and is based on the final-state topology of $${\mathrm{t}}\overline{{\mathrm{t}}}$$ events. The top quark decays almost exclusively into a W boson and a b quark, and only the subsequent decays of one or two of the W bosons into a charged lepton (electron or muon) and a neutrino are considered. These signatures imply the presence of isolated leptons with high transverse momentum $$p_{\mathrm {T}}$$, large $$p_{\mathrm {T}}$$ imbalance caused by the neutrinos that escape detection, and highly energetic jets. The identification of b jets through b-tagging techniques is used to increase the purity of the selected sample. The event selection in each channel is optimized to maximize the content of $${\mathrm{t}}\overline{{\mathrm{t}}}$$ signal events and background rejection.

### Lepton, jet, and missing transverse energy reconstruction

Events are reconstructed using a particle-flow technique [34, 35], which combines signals from all subdetectors to enhance the reconstruction and identification of individual particles observed in pp collisions. Charged hadrons from pileup events, i.e. those originating from additional pp interactions within the same bunch crossing, are subtracted on an event-by-event basis. Subsequently, the remaining neutral-hadron component from pileup is accounted for through jet energy corrections [36].

Electron candidates are reconstructed from a combination of the track momentum at the main interaction vertex, the corresponding energy deposition in the ECAL, and the energy sum of all bremsstrahlung photons attached to the track [37]. The candidates are required to have $p_{\mathrm {T}} > 33\,\text {GeV}$ within the pseudorapidity interval $|\eta | < 2.1$ for the $\ell +$jets channels, while electron candidates in the dilepton channels are required to have $p_{\mathrm {T}} > 20\,\text {GeV}$ and $|\eta | < 2.4$. As an additional quality criterion, a relative isolation $I_{\text {rel}}(0.3) < 0.10$ in the $\ell +$jets channels and $I_{\text {rel}}(0.3) < 0.15$ in the dilepton channels is required, where $I_{\text {rel}}(x)$ is defined as the sum of the $p_{\mathrm {T}}$ of all neutral and charged reconstructed particle candidates inside a cone of $\Delta R\equiv \sqrt{{(\Delta \eta )^2 + (\Delta \phi )^2}} < x$ around the electron (excluding the electron itself) in $\eta$-$\phi$ space, divided by the $p_{\mathrm {T}}$ of the electron.

Muon candidates are reconstructed using the track information from the silicon tracker and the muon system. They are required to have $p_{\mathrm {T}} > 33\,\text {GeV}$ and $|\eta | < 2.1$ in the $\ell +$jets channels, while in the dilepton channels the corresponding selection requires $p_{\mathrm {T}} >20\,\text {GeV}$ and $|\eta |<2.4$. Isolated muon candidates are selected if they fulfill $I_{\text {rel}}(0.4) < 0.12$ and $I_{\text {rel}}(0.3) < 0.15$ in the $\ell +$jets and dilepton channels, respectively. The same definition of relative isolation described above is also used for muon candidates.

Jets are reconstructed by clustering the particle-flow candidates [38] using the anti-$k_{\mathrm {T}}$ clustering algorithm with a distance parameter of $R = 0.5$ [39]. Electrons and muons passing less stringent selections on lepton kinematic quantities and isolation, relative to the ones specified above, are identified but excluded from clustering. A jet is selected if it has $p_{\mathrm {T}} > 30\,\text {GeV}$ and $|\eta | < 2.4$ for both the $\ell +$jets and dilepton channels. Jets originating from b quarks are identified through a “combined secondary vertex” algorithm [40], which provides a b-tagging discriminant by combining secondary vertices and track-based lifetime information. The chosen working point in the $\ell +$jets channels has an efficiency for tagging a b jet of $\approx$60 %, while the probability to misidentify light-flavour jets as b jets (mistag rate) is only ${\approx }$1.5 %. In the dilepton channels, the working point is selected to provide b-tagging efficiency and mistag rate of ${\approx }$80–85 and ${\approx }$10 %, respectively [40]. These requirements are chosen to reduce the background contribution in the corresponding channels while keeping a large fraction of the ${\mathrm{t}}\overline{{\mathrm{t}}}$ signal.

The missing transverse energy $E_{\mathrm {T}}/$ is defined as the magnitude of the imbalance in the transverse momentum $\mathbf {p_{\mathrm {T}}/}$ in the event, which is the negative of the vectorial sum of the momenta in the transverse plane of all the particles reconstructed with the particle-flow algorithm [41]. To mitigate the effect of contributions from pileup on the resolution in $E_{\mathrm {T}}/$, we use a multivariate correction where the input is separated into components that originate from the primary and other collision vertices [42]. This correction improves the $E_{\mathrm {T}}/$ resolution by $\approx$5 %.

### Event selection

Events in the $\ell +$jets channels that are triggered by the presence of a single electron (muon) with $p_{\mathrm {T}} > 27\,\text {GeV}$ ($p_{\mathrm {T}} > 24\,\text {GeV}$, $|\eta | < 2.1$), are selected if they contain exactly one reconstructed lepton fulfilling the requirements described in Sect. 4.1. Events are rejected if there are additional electron candidates with $p_{\mathrm {T}} > 20\,\text {GeV}$, $|\eta | < 2.5$, and $I_{\text {rel}}(0.3) < 0.15$, or additional muon candidates with $p_{\mathrm {T}} > 10\,\text {GeV}$, $|\eta | < 2.5$, and $I_{\text {rel}}(0.4) < 0.2$. Additionally, an event must contain at least four reconstructed jets satisfying the criteria described in Sect. 4.1. To suppress background contribution mainly from W$+$jets events, at least two of these jets are required to be tagged as b jets, and at least two must not be tagged as b jets, as they are used to reconstruct $\mathrm {W}\rightarrow {\mathrm{q}} \overline{{\mathrm{q}}} '$ decays. In the dilepton channels, events are triggered using combinations of two leptons with $p_{\mathrm {T}}$ thresholds of 8 and 17$\,\text {GeV}$, and are selected if they contain at least two isolated leptons of opposite electric charge and at least two jets. At least one of the jets is required to be b-tagged. In events with more than two leptons, we choose the lepton pair with opposite charge and largest value in the sum of their scalar $p_{\mathrm {T}}$. Events with an invariant mass of the lepton pair smaller than 20$\,\text {GeV}$ are removed to suppress events from decays of heavy-flavour resonances and low-mass Drell–Yan processes. Backgrounds from Z$+$jets processes in the $\mathrm {e}^+\mathrm {e}^-$ and $\mu ^+ \mu ^-$ channels are also suppressed by requiring the dilepton invariant mass to be outside a Z boson mass window of $91 \pm 15\,\text {GeV}$, and to have $E_{\mathrm {T}}/> 40\,\text {GeV}$.

**Fig. 1:**
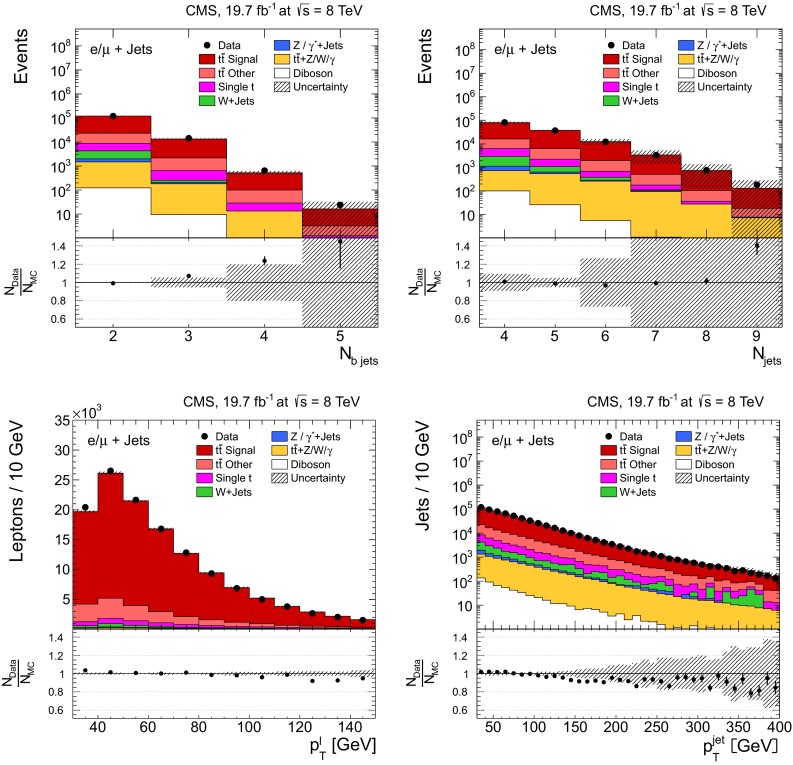
Kinematic distributions after event selection and before the kinematic reconstruction of the ${\mathrm{t}}\overline{{\mathrm{t}}}$ system in the $\ell +$jets channels: the multiplicity in the reconstructed number of b-tagged jets (*top left*), the multiplicity in the reconstructed number of jets (*top right*), the $p_{\mathrm {T}}$ of the selected isolated leptons (*bottom left*), and the $p_{\mathrm {T}}$ of all reconstructed jets (*bottom right*). The QCD multijet background is negligible and not shown. The *hatched regions* correspond to the *shape* uncertainties for the signal and backgrounds (cf. Sect. 5). The *lower part of each plot* shows the ratio of data to the predictions

**Fig. 2:**
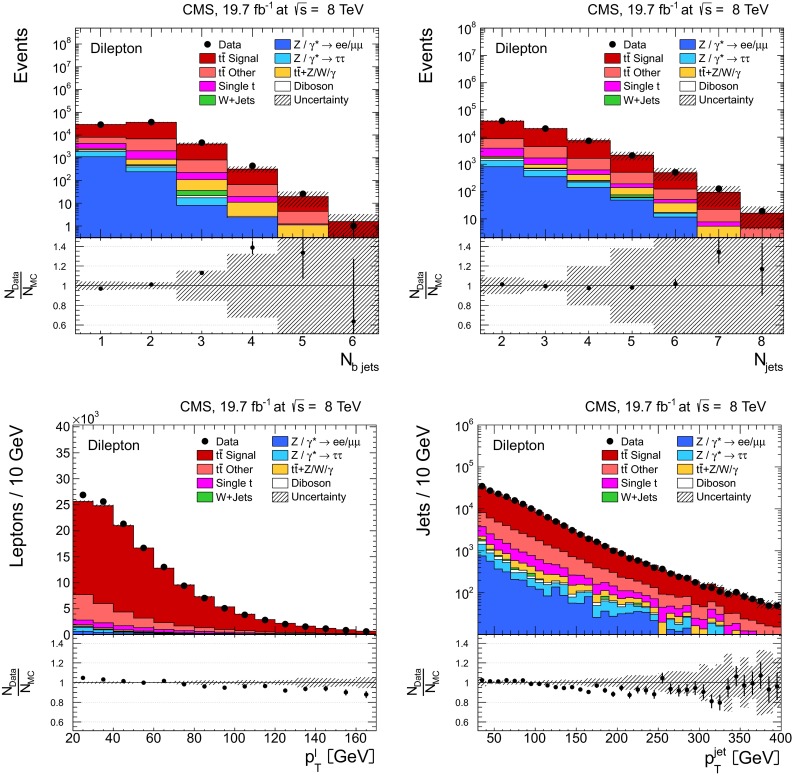
Kinematic distributions after event selection and before the kinematic reconstruction of the ${\mathrm{t}}\overline{{\mathrm{t}}}$ system for the dilepton channels: the multiplicity in the reconstructed number of b-tagged jets (*top left*), the multiplicity in the number of reconstructed jets (*top right*), the $p_{\mathrm {T}}$ of the selected isolated leptons (*bottom left*), and the $p_{\mathrm {T}}$ of the reconstructed jets (*bottom right*). The QCD multijet background is negligible and not shown. The Z/$\gamma ^{*}$ $+$jets background is determined from data [5, 43]. The *hatched regions* correspond to the *shape* uncertainties for the signal and backgrounds (cf. Sect. 5). The *lower part of each plot* shows the ratio of data to the predictions

After these selection steps, several basic distributions in $\ell +$jets and dilepton events are shown in Figs. 1 and 2, respectively. The hatched regions correspond to the shape uncertainties for the signal and background (cf. Sect. 5), and are dominated by the former. The data are reasonably well described by the simulation, as shown in the lower part of each plot, where the ratio of data to simulation is presented to better indicate the level of agreement between data and the default ${\mathrm{t}}\overline{{\mathrm{t}}}$ signal (MadGraph$+$pythia 6) and background samples used in the analysis. For both channels, however, data tend to have lower $p_{\mathrm {T}}$ values than predicted by the simulation. It has been verified that the results presented in Sect. 6 are not affected by these remaining differences between data and simulation. A better data-to-simulation agreement in the lepton and jet $p_{\mathrm {T}}$ distributions is obtained by scaling the top quark $p_{\mathrm {T}}$ spectrum in simulation to match the data. However, the impact on the measurement of the cross sections is negligible.

### Kinematic reconstruction of the ${\mathrm{t}}\overline{{\mathrm{t}}}$ system

The kinematic properties of the top quark pair are determined from the four-momenta of all final-state objects through kinematic reconstruction algorithms. These algorithms are improved versions of those described in Ref. [5].

In the $\ell +$jets channels, a constrained kinematic fitting algorithm is applied [5, 44] to the four-momenta of the selected lepton and up to five leading jets, and the $\mathbf {p_{\mathrm {T}}/}$ representing the transverse momentum of the neutrino, which are changed according to their resolutions. The fit is constrained to reconstruct two W bosons, each with a mass of $80.4\,\text {GeV}$. In addition, the reconstructed top quark and antiquark masses are required to be equal. To reduce the number of permutations in the association of jets to quarks, only b-tagged jets are considered as b quarks, and only untagged jets are considered as light quarks. In events with several combinatorial solutions, only the one with the minimum $\chi ^{2}$ in the fit is accepted. The main improvement relative to the method described in Ref. [5] is the increase in the number of correct assignments of b jets to b quarks. This is achieved by applying the kinematic fit twice, sequentially, in each event. In the first fit, the top quark mass is fixed to a value of 172.5$\,\text {GeV}$. The jet combination that provides the minimum $\chi ^{2}$ in the fit is then used as input to the second kinematic fit, in which the top quark mass is not fixed, and the solution to this fit is retained. A further improvement in the method is to require the $\chi ^{2}$-probability of the second kinematic fit to be $>$2 %. This criterion is chosen to optimize the fraction of correctly reconstructed signal events, without increasing significantly the statistical uncertainty in the data. The efficiency of this requirement is about 87 % for signal events with the correct jet assignment. As a result, the number of correctly reconstructed events is increased by almost a factor of two relative to the method used in Ref. [5], and effects from migration of events across bins, which are relevant for the measurements of the cross section, are reduced. It has been checked that any possible bias in the results that could be introduced by fixing the top quark mass to a specific value in the first kinematic fit is within the assigned systematic uncertainty on the dependence of the measurement on the top quark mass (cf. Sect. 5.2).

The dilepton channels use an algebraic kinematic reconstruction method [5, 45]. The only unknowns are the three-momenta of the two neutrinos, which are reconstructed imposing the following kinematic constraints: $p_{\mathrm {T}}$ conservation in the event; the W bosons, and top quark and antiquark masses. In contrast to the method of Ref. [5], the top quark mass is fixed to a value of 172.5$\,\text {GeV}$. Each suitable pair of b jet candidates in the event, and both possible assignments of these two jets to the two selected leptons, are considered in the kinematic reconstruction. Combinations with two b-tagged jets are preferred to using single b-tagged jets. In the new method, events are reconstructed 100 times, each time randomly smearing the measured energies and directions of the reconstructed lepton and b jet candidates by their respective detector resolutions. This smearing recovers events that yielded no solution of the equations for the neutrino momenta, because of measurement fluctuations. The equations for the neutrino momenta can have up to four solutions. For a given smearing, the solution is identified by the one yielding the smallest invariant mass of the ${\mathrm{t}}\overline{{\mathrm{t}}}$ system. For each solution, a weight is calculated based on the expected true lepton-b-jet invariant mass spectrum. The weights are summed over the 100 reconstruction attempts, and the kinematic quantities associated to the top quark and antiquark are calculated as a weighted average. Finally, the two jet and lepton-jet assignments that yield the maximum sum of weights are chosen for analysis. It has been checked that any bias introduced through the use of the lepton-b-jet and ${\mathrm{t}}\overline{{\mathrm{t}}}$ invariant masses is negligible. This method yields on average a reconstruction efficiency of $\approx$94 %, which is 6 % higher than the one described in Ref. [5], and reduces systematic migration effects.

**Fig. 3:**
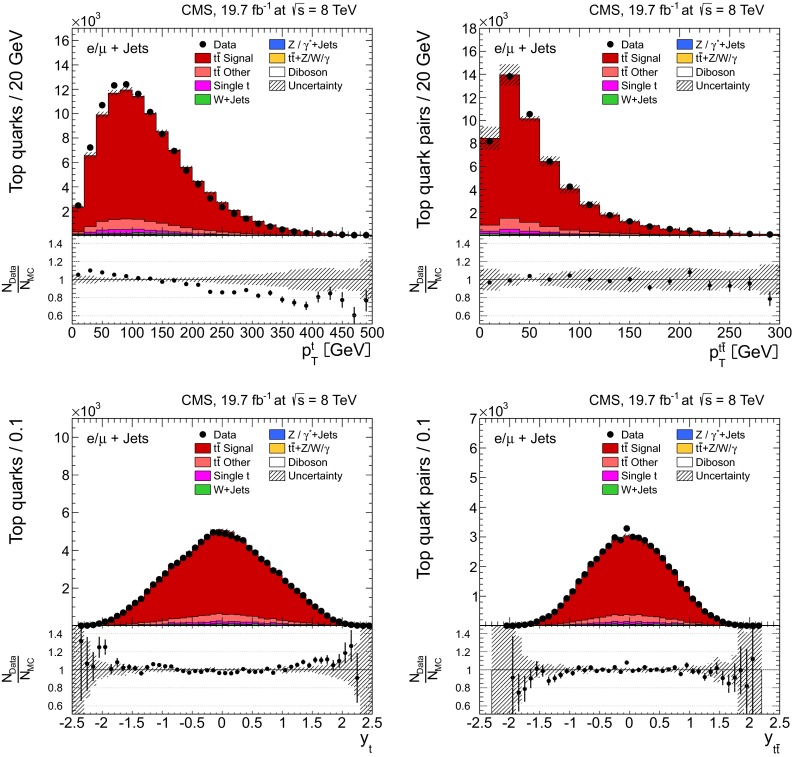
Distribution of top quark or antiquark (*left*) and ${\mathrm{t}}\overline{{\mathrm{t}}}$ (*right*) quantities as obtained from the kinematic reconstruction in the $\ell +$jets channels. The *top row* shows the $p_{\mathrm {T}}$, and the *bottom row* shows the rapidities. The QCD multijet background is negligible and not shown. The *hatched regions* correspond to the *shape* uncertainties for the signal and backgrounds (cf. Sect. 5). The *lower part of each plot* shows the ratio of data to the predictions

**Fig. 4:**
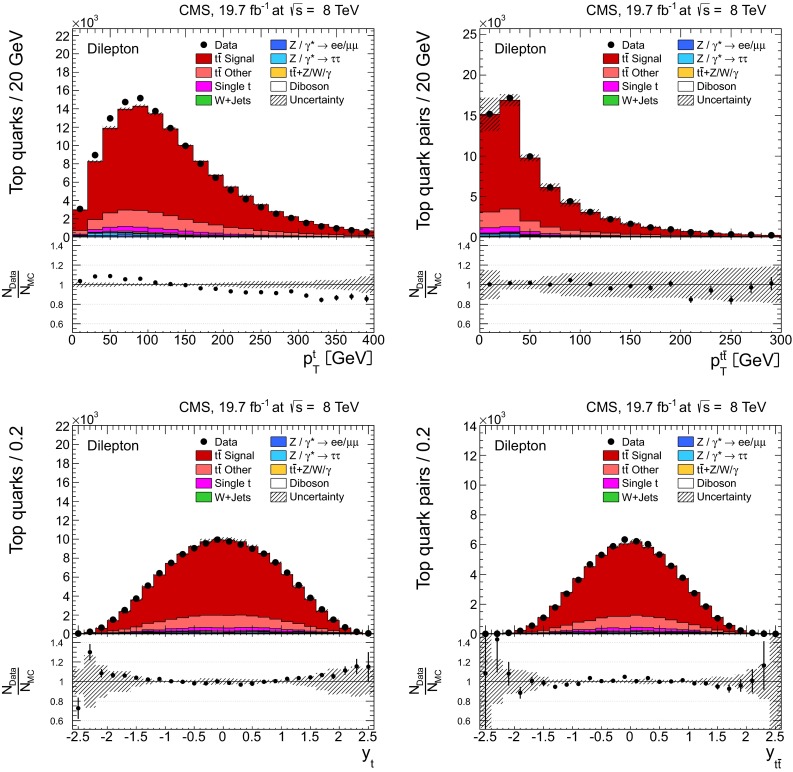
Distribution of top quark or antiquark (*left*) and ${\mathrm{t}}\overline{{\mathrm{t}}}$ (*right*) quantities as obtained from the kinematic reconstruction in the dilepton channels. The *top row* shows the $p_{\mathrm {T}}$, and the *bottom row* shows the rapidities. The QCD multijet background is negligible and not shown. The Z/$\gamma ^{*}$ $+$jets background is determined from data [5, 43]. The *hatched regions* correspond to the *shape* uncertainties for the signal and backgrounds (cf. Sect. 5). The *lower part of each plot* shows the ratio of data to the predictions

Distributions of the top quark or antiquark and ${\mathrm{t}}\overline{{\mathrm{t}}}$ kinematic observables (the transverse momenta $p_{\mathrm {T}} ^{{\mathrm{t}}}$, $p_{\mathrm {T}} ^{{\mathrm{t}}\overline{{\mathrm{t}}}}$, and the rapidities $y_{{\mathrm{t}}}$ and $y_{{\mathrm{t}}\overline{{\mathrm{t}}}}$) are presented in Figs. 3 and 4 for the $\ell +$jets and dilepton channels, respectively. The hatched regions correspond to the shape uncertainties for the signal and background (cf. Sect. 5), and are dominated by the former. The lower panel in each plot also shows the ratio of data relative to the simulated signal and background samples.

In general, the data are reasonably well described by the simulation within the uncertainties. For both channels, the measured $$p_{\mathrm {T}}$$ distributions, in particular $$p_{\mathrm {T}} ^{{\mathrm{t}}}$$, are somewhat softer than the simulated distributions: the data lie above the simulation for $$p_{\mathrm {T}} ^{{\mathrm{t}}} < 60 (65)$$$$\,\text {GeV}$$ in the $$\ell +$$jets (dilepton) channels, while they lie below for $$p_{\mathrm {T}} ^{{\mathrm{t}}} > 200$$$$\,\text {GeV}$$. This pattern was also observed at 7$$\,\text {TeV}$$  [5]. To ensure that the results presented in Sect. 6 are not affected by such small remaining differences between data and simulation, the analysis has been repeated in different kinematic regions, with different selection requirements, and after scaling the top quark $$p_{\mathrm {T}}$$ spectrum in simulation to match the data. However, the impact on the measurement of the cross sections is negligible.

Following the event selection described in Sect. 4.2 and the kinematic reconstruction of the $${\mathrm{t}}\overline{{\mathrm{t}}}$$ system, the main contributions to the background in the $$\ell +$$jets channels arise from $${\mathrm{t}}\overline{{\mathrm{t}}}$$ decays into channel other than $$\ell +$$jets (including $${\mathrm{t}}\overline{{\mathrm{t}}}$$ decays into $$\tau $$ leptons originating from the primary interaction) and single top quark events. The contribution from W$$+$$jets and QCD multijet events are well suppressed after the b-tagging requirement, while other $${\mathrm{t}}\overline{{\mathrm{t}}}$$ events are somewhat reduced after the $$\chi ^{2}$$-probability requirement. A total of 24,927 events are found in the $$\mathrm {e}$$$$+$$jets channel and 26,843 events in the $$\mathrm {\mu }$$$$+$$jets channel. The contribution from $${\mathrm{t}}\overline{{\mathrm{t}}}$$ signal to the final event sample is 89.0 %. The remaining fraction of events contains 7.3 % $${\mathrm{t}}\overline{{\mathrm{t}}}$$ decays other than the $$\ell +$$jets channels, 2.4 % single top quark events, 0.9 % W$$+$$jets and $${\mathrm{t}}\overline{{\mathrm{t}}}+\text{Z/W/}{\gamma}$$ events, and negligible fractions of Z$$+$$jets, diboson, and QCD multijet events. All background contributions are determined from simulation.

In the dilepton channels, 10,678 events are found in the $\mathrm {e}^+\mathrm {e}^-$ channel, 14,403 in the $\mu ^+ \mu ^-$ channel, and 39,640 in the $\mathrm {e}^\pm \mu ^{\mp }$ channel. Only ${\mathrm{t}}\overline{{\mathrm{t}}}$ events containing at least two leptons (electrons or muons) from W decays in the final state are considered as signal, and constitute 79.0 % of the final event sample. All other ${\mathrm{t}}\overline{{\mathrm{t}}}$ candidate events, specifically those originating from decays via $\tau$ leptons, are considered as background and amount to 13.3 % of the final event sample. The fraction of Z$+$jets events is found to be 2.4 %. This background, which is dominant to the $\mathrm {e}^+\mathrm {e}^-$ and $\mu ^+ \mu ^-$ channels, is estimated from data using the number of events observed within the Z-peak region (which is removed from the candidate sample), and a correction needed for non-Z$+$jets backgrounds in this same control region is obtained from data in the $\mathrm {e}^\pm \mu ^{\mp }$ channel [5, 43]. Other sources of background, including single top quark production (3.4 %), ${\mathrm{t}}\overline{{\mathrm{t}}}+\text{Z/W/}{\gamma}$ production (1 %), the contribution arising from misidentified or genuine leptons within jets (0.6 %), or diboson events (0.3 %), are estimated from simulation.

## Systematic uncertainties

The measurement is affected by systematic uncertainties that originate from detector effects and from theoretical assumptions. Each source of systematic uncertainty is assessed individually by changing the corresponding efficiency, resolution, or scale by its uncertainty, using a prescription similar to the one followed in Ref. [5]. For each change made, the measured normalized differential cross section is recalculated, and the difference of the changed result relative to its nominal value in each bin is taken as the systematic uncertainty. The overall uncertainty on the measurement is obtained by adding all the contributions in quadrature, and is of the order of 3–10 %, depending on the observable and the bin. A detailed description of this is given in Sects. 5.1 and 5.2. The typical representative values of the systematic uncertainties in the normalized differential cross sections are summarized in Table 1.

### Experimental uncertainties

The efficiencies of the single-electron and single-muon triggers in the $\ell +$jets channels are determined using the “tag-and-probe” method of Ref. [46] using Z boson event samples. Scale factors close to unity within a few percent are extracted to account for the observed dependence on the $\eta$ and $p_{\mathrm {T}}$ of the lepton. The lepton identification and isolation efficiencies for the $\ell +$jets channels obtained with the tag-and-probe method agree well between data and simulation, so that the applied corrections are very close to unity. The systematic uncertainties are determined by shape-dependent changes in trigger and selection efficiencies by their uncertainties. Lepton trigger efficiencies in the dilepton channels are measured using triggers that are only weakly correlated to the dilepton triggers used in the analysis. A dependence on $\eta$ of a few percent is observed, and scale factors are extracted. The lepton identification and isolation uncertainties in the dilepton channels are also determined using the tag-and-probe method, and are again found to be described very well by the simulation for both electrons and muons. The overall difference between data and simulation in bins of $\eta$ and $p_{\mathrm {T}}$ is estimated to be $<$2 % for electrons, and scale factors for muons are found to be close to unity within 1.0 %.

The uncertainty due to the limited knowledge of the jet energy scale is determined by changes implemented in jet energy in bins of $p_{\mathrm {T}}$ and $\eta$ [38]. The uncertainty due to the limited accuracy of the jet energy resolution (JER) is determined by changing the simulated JER by ${\pm }1\sigma$ in different $\eta$ regions [38].

The uncertainty in b-tagging efficiency is determined by taking the maximum change in the shape of $p_{\mathrm {T}}$ and $\eta$ b jet distributions obtained by changing the scale factors. This is achieved by dividing the b jet distributions in $p_{\mathrm {T}}$ and $\eta$ into two bins at the median of the respective distributions. These correspond to $p_{\mathrm {T}} =65\,\text {GeV}$, and $|\eta |=0.7$ and 0.75 for the $\ell +$jets and dilepton channels, respectively. The b-tagging scale factors for b jets in the first bin are scaled up by half of the uncertainties quoted in Ref. [40], while those in the second bin are scaled down, and vice versa, so that a maximum variation is assumed and the difference between the scale factors in the two bins reflects the full uncertainty. The changes are made separately in the $p_{\mathrm {T}}$ and $\eta$ distributions, and independently for heavy-flavour (b and c) and light (s, u, d, and gluon) jets, assuming that they are all uncorrelated.

The uncertainty in background normalization is determined by changing the background yields. In the $\ell +$jets channels, the background normalization for the diboson, QCD multijet, W$+$jets, and Z$+$jets samples is conservatively varied by $\pm$50 % [5], since these backgrounds, being very small, are determined from simulation rather than from data. The normalization of the ${\mathrm{t}}\overline{{\mathrm{t}}}+\text{Z/W/}{\gamma}$ samples is changed by $\pm$30 %. For the single top quark sample, the uncertainty is covered by changing the normalization by $\pm$30 %, and the kinematic scales of the event process (renormalization and factorization scales) as described in Sect. 5.2. In the $\mathrm {e}^+\mathrm {e}^-$ and $\mu ^+ \mu ^-$ channels, the dominant background from Z$+$jets determined from data [5, 43] is changed in normalization by $\pm$30 %. In addition, changes in the background contributions from single top quark, diboson, QCD multijet, ${\mathrm{t}}\overline{{\mathrm{t}}}+\text{Z/W/}\gamma$, and W$+$jets events of $\pm$30 % are used in dilepton channels [5].

The kinematic reconstruction of top quarks is well described by the simulation, and the resulting uncertainties are small. In the case of the $\ell +$jets analysis, the uncertainty of the kinematic fit is included in the changes in jet energy scales and resolutions, and in the uncertainty on the dependence on the top quark mass (cf. Sect. 5.2). In the dilepton analysis, the bin-to-bin uncertainty is determined from the small remaining difference in efficiency between simulation and data.

The pileup model estimates the mean number of additional pp interactions to be about 20 events per bunch crossing for the analyzed data. This estimate is based on the total inelastic proton–proton cross section, which is determined to be 69.4 $\text {\,mb}$ following the measurement described in Ref. [47]. The systematic uncertainty is determined by changing this cross section within its uncertainty of $\pm$5 %.

### Uncertainties in modelling

The impact of theoretical assumptions on the measurement is determined, as indicated previously, by repeating the analysis and replacing the standard MadGraph${\mathrm{t}}\overline{{\mathrm{t}}}$ simulation by dedicated simulation samples with altered parameters.

The uncertainty in modelling of the hard-production process is assessed through changes in the renormalization and factorization scales in the MadGraph sample by factors of two and 0.5 relative to their common nominal value, which is set to the *Q* of the hard process. In MadGraph, *Q* is defined by $Q^2 = m^2_{{\mathrm{t}}} + \Sigma p_{\mathrm {T}} ^2$, where the sum is over all additional final state partons in the matrix element. The impact of the choice of the scale that separates the description of jet production through matrix elements (ME) or parton shower (PS) in MadGraph is studied by changing its reference value of 20 $\,\text {GeV}$ to 40 and 10$\,\text {GeV}$. In the $\ell +$jets channels, changes in the renormalization and factorization scales are also applied to single top quark events to determine an uncertainty on the shape of this background contribution. The dependence of the measurement on the top quark mass is also estimated from dedicated MadGraph simulation samples in which the top quark mass is changed by $\pm$1$\,\text {GeV}$ relative to the value used in the default simulation. The uncertainty from hadronization and parton showering is assessed by comparing the results obtained from samples simulated with powheg and mc@nlo interfaced with pythia 6 and herwig 6, respectively. The uncertainty from the choice of PDF is determined by reweighting the sample of simulated ${\mathrm{t}}\overline{{\mathrm{t}}}$ signal events according to the 52 CT10 PDF error sets [24], at a 90 % confidence level. The maximum variation is taken as uncertainty. As mentioned in Sects. 4.2 and 4.3, the effect of scaling the top quark $p_{\mathrm {T}}$ spectrum in simulation to match the data has negligible impact on the measured cross sections, therefore no systematic uncertainty is taken into account for this effect.

**Table 1:** Breakdown of typical systematic uncertainties for the normalized differential cross sections. The uncertainty in the jet-parton matching threshold is indicated as “ME-PS threshold”; “PS” refers to “parton shower”. The medians of the distribution of uncertainties over all bins of the measurement are quoted. For the $\ell +$jets channels, the background from Z$+$jets is negligible and included in the “Background (all other)” category

Source	Relative systematic uncertainty (%)			
	Lepton and b jet observables		Top quark and ${\mathrm{t}}\overline{{\mathrm{t}}}$ observables	
	$\ell +$jets	Dileptons	$\ell +$jets	Dileptons
Trigger eff. and lepton selec.	0.1	0.1	0.1	0.1
Jet energy scale	2.3	0.4	1.6	0.8
Jet energy resolution	0.4	0.2	0.5	0.3
Background (Z$+$jets)	–	0.2	–	0.1
Background (all other)	0.9	0.4	0.7	0.4
b tagging	0.7	0.1	0.6	0.2
Kinematic reconstruction	–	${<}0.1$	–	${<}0.1$
Pileup	0.2	0.1	0.3	0.1
Fact./renorm. scale	1.1	0.7	1.8	1.2
ME-PS threshold	0.8	0.5	1.3	0.8
Hadronization and PS	2.7	1.4	1.9	1.1
Top quark mass	1.5	0.6	1.0	0.7
PDF choice	0.1	0.2	0.1	0.5

## Normalized differential cross sections

The normalized ${\mathrm{t}}\overline{{\mathrm{t}}}$ cross section in each bin *i* of each observable *X* is determined as a function of the kinematic properties of the leptons, the lepton pair, the b jets, the b jet system, the top quarks, and the ${\mathrm{t}}\overline{{\mathrm{t}}}$ system through the relation [5]:

1 $$\begin{aligned} \frac{1}{\sigma }\frac{{\mathrm{d}}\sigma _{i}}{{\mathrm{d}}X}=\frac{1}{\sum _{i}x_{i}}\frac{x_{i}}{\Delta ^{\text {X}}_{i}} \end{aligned}$$

where $x_{i}$ represents the number of signal events measured in data in bin *i* after background subtraction and corrected for detector efficiencies, acceptances, and migrations, and $\Delta _{i}^{X}$ is the bin width. The differential cross section is normalized by the sum of $x_i$ over all bins, as indicated in Eq. (1). The integrated luminosity is omitted, as it cancels in the ratio. Because of the normalization, sources of systematic uncertainty that are correlated across all bins of the measurement, e.g. the uncertainty in the integrated luminosity, also cancel. The contribution to the background from other ${\mathrm{t}}\overline{{\mathrm{t}}}$ decays is taken into account, after subtracting all other background components, by correcting the number of signal events in data using the expected signal fraction. The expected signal fraction is defined as the ratio of the number of selected ${\mathrm{t}}\overline{{\mathrm{t}}}$ signal events to the total number of selected ${\mathrm{t}}\overline{{\mathrm{t}}}$ events (i.e. signal and all other ${\mathrm{t}}\overline{{\mathrm{t}}}$ events) in simulation. This procedure avoids the dependence on the total inclusive ${\mathrm{t}}\overline{{\mathrm{t}}}$ cross section used in the normalization of the simulated signal sample.

Effects from trigger and detector efficiencies and resolutions leading to the migration of events across bin boundaries, and therefore to statistical correlations among neighbouring bins, are corrected by using a regularized unfolding method [5, 48, 49]. For each measured distribution, a response matrix is defined that accounts for migrations and efficiencies using the simulated MadGraph$+$pythia 6 ${\mathrm{t}}\overline{{\mathrm{t}}}$ signal sample. The generalized inverse of the response matrix is used to obtain the unfolded distribution from the measured distribution by applying a $\chi ^2$ minimization technique. A smoothing prescription (regularization) is applied to prevent large unphysical fluctuations that can be introduced when directly inverting the response matrix. The strength of the regularization is determined and optimized individually for each distribution using the averaged global correlation method [50]. To keep the bin-to-bin migrations small, the widths of bins in the measurement are chosen according to their purity (ratio of the number of events generated and reconstructed in a particular bin to the total number of events reconstructed in that bin; this quantity is sensitive to migrations into the bin) and stability (ratio of the number of events generated and reconstructed in a particular bin to the number of events generated in that bin; this is sensitive to migrations out of the bin). The purity and stability of the bins in this analysis are typically 60 % or larger, mainly due to the improvements in the kinematic reconstruction methods discussed in Sect. 4.3.

The performance of the unfolding procedure is tested for possible biases from the choice of the input model (the MadGraph$+$pythia 6 ${\mathrm{t}}\overline{{\mathrm{t}}}$ signal simulation). It is verified that, either by reweighting the signal simulation or injecting a resonant ${\mathrm{t}}\overline{{\mathrm{t}}}$ peak into the simulation of the signal, the unfolding procedure based on the nominal response matrices still recovers these altered shapes within statistical uncertainties. Moreover, ${\mathrm{t}}\overline{{\mathrm{t}}}$ samples simulated with powheg$+$pythia 6 and mc@nlo$+$herwig 6 are used to obtain the response matrices applied in the unfolding when determining the systematic uncertainties of the model (cf. Sect. 5.2). Therefore, possible effects from the unfolding procedure are already taken into account in the systematic uncertainties. The unfolded results are found to be consistent with those obtained using other regularization techniques [49].

The measurement of the normalized differential cross sections proceeds as follows. For each kinematic distribution, the event yields in the separate channels are added together, the background is subtracted, and the unfolding is performed. It is verified that the measurements in separate channels yield results consistent within their uncertainties. The systematic uncertainties in each bin are determined from the changes in the combined cross sections. This requires the full analysis to be repeated for every systematic change, and the difference relative to the nominal combined value is taken as the systematic uncertainty for each bin of each observable. This method therefore takes into account the correlation among systematic uncertainties in different channels and bins.

The normalized differential cross sections of leptons and b jets are unfolded to the particle level and determined in a fiducial phase space defined by the kinematic and geometric region in which the final-state leptons and jets are produced within the detector acceptance (cf. Sect. 6.1). This minimizes model uncertainties from the extrapolation of the measurement outside of the experimentally well-described regions of phase space. In addition, the top quark and ${\mathrm{t}}\overline{{\mathrm{t}}}$-system quantities are unfolded to the parton level and presented in the full phase space (cf. Sect. 6.2) to provide easier comparisons with recent QCD calculations. The measurements are compared to predictions from MadGraph$+$pythia 6, powheg$+$pythia 6, powheg$+$herwig 6, and mc@nlo$+$herwig 6. The top quark and ${\mathrm{t}}\overline{{\mathrm{t}}}$ results are also compared to the latest calculations at NLO$+$NNLL [14, 15] and approximate NNLO [16] precision, when available.

In addition to the measurements discussed in Ref. [5], results for the $p_{\mathrm {T}}$ and invariant mass of the b jet pair, the $p_{\mathrm {T}}$ of the top quarks or antiquarks in the ${\mathrm{t}}\overline{{\mathrm{t}}}$ rest frame, the $p_{\mathrm {T}}$ of the highest (*leading*) and second-highest (*trailing*) $p_{\mathrm {T}}$ of the top quark or antiquark, and the difference in the azimuthal angle between the top quark and antiquark are also presented.

All values of normalized differential cross sections, including bin boundaries, are provided in tables in the supplemental material (URL will be inserted by publisher)

### Lepton and b jet differential cross sections

The normalized differential ${\mathrm{t}}\overline{{\mathrm{t}}}$ cross section as a function of the lepton and b jet kinematic properties is measured at the particle level, where the objects are defined as follows. Leptons from W boson decays are defined after final-state radiation. A jet is defined at the particle level, following a procedure similar to that described in Sect. 4.1 for reconstructed jets, by applying the anti-$k_{\mathrm {T}}$ clustering algorithm with a distance parameter of 0.5 to all stable particles (excluding the decay products from W boson decays into e$\nu$, $\mu \nu$, and final states with leptonic $\tau$ decays). A jet is defined as a b jet if it contains any of the decay products of a B hadron. Only the two b jets of highest $p_{\mathrm {T}}$ originating from different B hadrons are considered as arising from the top quark decays.

The measurements are presented in a fiducial phase space defined by geometric and kinematic requirements on these particle-level objects as follows. The charged leptons from the W boson decays must have $|\eta |<2.1$ and $p_{\mathrm {T}} > 33\,\text {GeV}$ in the $\ell +$jets channels, and $|\eta | < 2.4$ and $p_{\mathrm {T}} > 20\,\text {GeV}$ in the dilepton channels. Exactly one and two leptons are required, respectively, in the $\ell +$jets and the dilepton channels. At least four jets with $|\eta | < 2.4$ and $p_{\mathrm {T}} > 30\,\text {GeV}$, two of which are b jets, are required in the $\ell +$jets channels. In the dilepton channels, both b jets from the top quark decays must satisfy $|\eta | < 2.4$ and $p_{\mathrm {T}} > 30\,\text {GeV}$. The fiducial particle-level corrections are determined using simulated ${\mathrm{t}}\overline{{\mathrm{t}}}$ events that fulfill these requirements; all other ${\mathrm{t}}\overline{{\mathrm{t}}}$ events are classified as background and are removed.

**Fig. 5:**
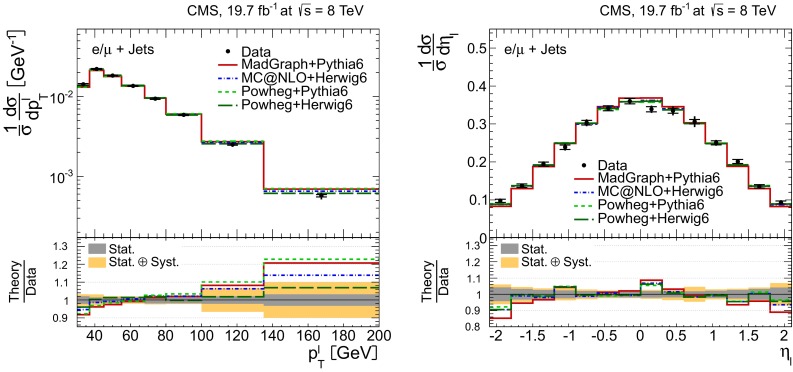
Normalized differential ${\mathrm{t}}\overline{{\mathrm{t}}}$ production cross section in the $\ell +$jets channels as a function of the $p_{\mathrm {T}} ^{\ell }$ (*left*) and $\eta _{\ell }$ (*right*) of the charged lepton. The *superscript* ‘$\ell$’ refers to both $\ell ^{+}$ and $\ell ^{-}$. The data points are placed at the midpoint of the bins. The *inner* (*outer*) *error bars* indicate the statistical (combined statistical and systematic) uncertainties. The measurements are compared to predictions from MadGraph $+$ pythia 6, powheg $+$ pythia 6, powheg $+$ herwig 6, and mc@nlo $+$ herwig 6. The *lower part of each plot* shows the ratio of the predictions to data

**Fig. 6:**
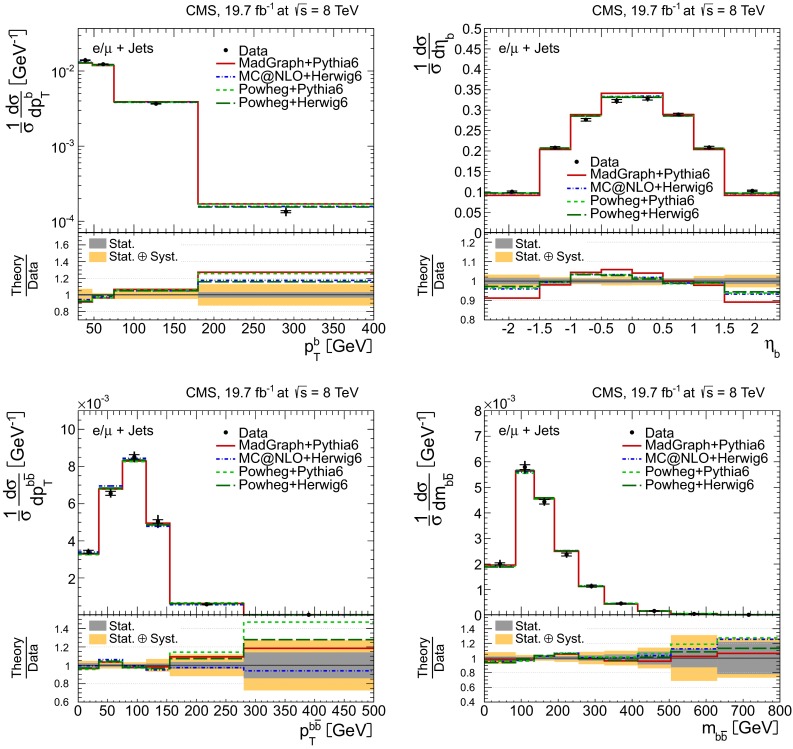
Normalized differential ${\mathrm{t}}\overline{{\mathrm{t}}}$ production cross section in the $\ell +$jets channels as a function of the $p_{\mathrm {T}} ^{{\mathrm{b}}}$ (*top left*) and $\eta _{{\mathrm{b}}}$ (*top right*) of the b jets, and the $p_{\mathrm {T}} ^{{\mathrm{b}} \overline{{\mathrm{b}}} }$ (*bottom left*) and $m_{{\mathrm{b}} \overline{{\mathrm{b}}} }$ (*bottom right*) of the b jet pair. The *superscript* ‘b’ refers to both b and $\overline{{\mathrm{b}}}$ jets. The data points are placed at the midpoint of the bins. The *inner* (*outer*) *error bars* indicate the statistical (combined statistical and systematic) uncertainties. The measurements are compared to predictions from MadGraph $+$ pythia 6, powheg $+$ pythia 6, powheg $+$ herwig 6, and mc@nlo $+$ herwig 6. The *lower part of each plot* shows the ratio of the predictions to data

Figure 5 presents the normalized differential cross section in the $\ell +$jets channels as a function of the lepton transverse momentum $p_{\mathrm {T}} ^{\ell }$ and pseudorapidity $\eta _{\ell }$. The distributions of the transverse momentum of the b jets $p_{\mathrm {T}} ^{{\mathrm{b}}}$ and their pseudorapidity $\eta _{{\mathrm{b}}}$ are given in Fig. 6, together with the transverse momentum $p_{\mathrm {T}} ^{{\mathrm{b}} \overline{{\mathrm{b}}} }$ and invariant mass $m_{{\mathrm{b}} \overline{{\mathrm{b}}} }$ of the b jet pair. Also shown are predictions from MadGraph$+$pythia 6, powheg$+$pythia 6, powheg$+$herwig 6, and mc@nlo$+$herwig 6. The lower panel in each plot shows the ratio of each of these predictions to data, in order to quantify their level of agreement relative to data.

**Fig. 7:**
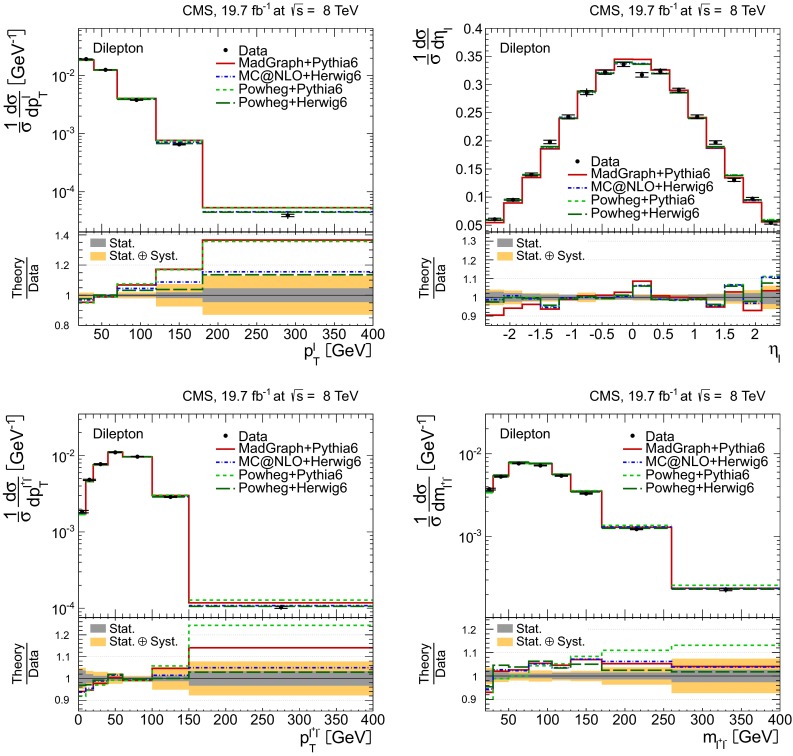
Normalized differential ${\mathrm{t}}\overline{{\mathrm{t}}}$ production cross section in the dilepton channels as a function of the $p_{\mathrm {T}} ^{\ell }$ (*top left*) and $\eta _{\ell }$ (*top right*) of the charged leptons, and the $p_{\mathrm {T}} ^{\ell ^{+}\ell ^{-}}$ (*bottom left*) and $m_{\ell ^{+}\ell ^{-}}$ (*bottom right*) of the lepton pair. The *superscript* ‘$\ell$’ refers to both $\ell ^{+}$ and $\ell ^{-}$. The data points are placed at the midpoint of the bins. The *inner* (*outer*) *error bars* indicate the statistical (combined statistical and systematic) uncertainties. The measurements are compared to predictions from MadGraph $+$ pythia 6, powheg $+$ pythia 6, powheg $+$ herwig 6, and mc@nlo $+$ herwig 6. The *lower part of each plot* shows the ratio of the predictions to data

**Fig. 8:**
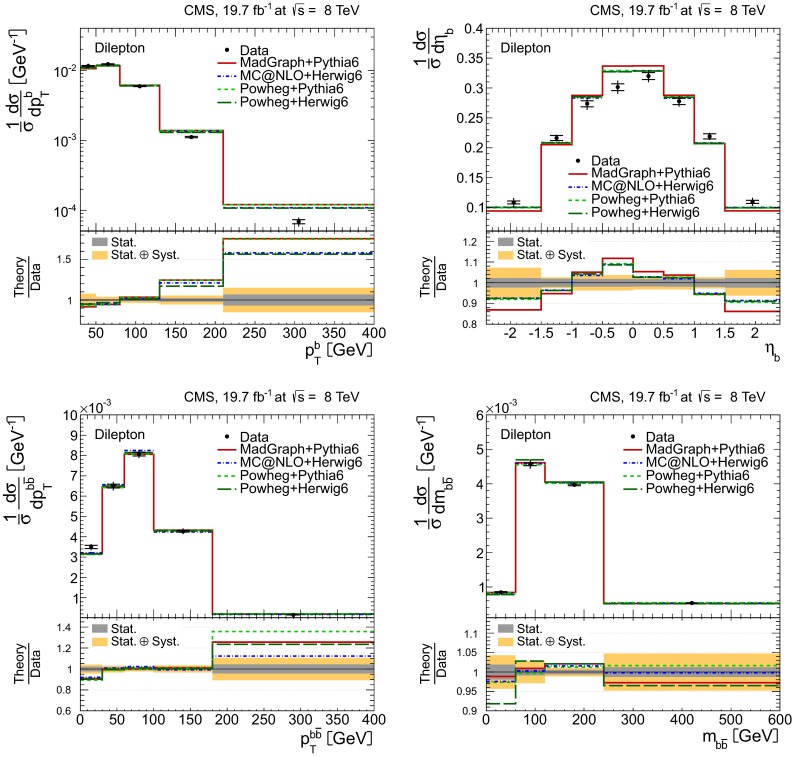
Normalized differential ${\mathrm{t}}\overline{{\mathrm{t}}}$ production cross section in the dilepton channels as a function of the $p_{\mathrm {T}} ^{{\mathrm{b}}}$ (*top left*) and $\eta _{{\mathrm{b}}}$ (*top right*) of the b jets, and the $p_{\mathrm {T}} ^{{\mathrm{b}} \overline{{\mathrm{b}}} }$ (*bottom left*) and $m_{{\mathrm{b}} \overline{{\mathrm{b}}} }$ (*bottom right*) of the b jet pair. The *superscript* ‘b’ refers to both b and $\overline{{\mathrm{b}}}$ jets. The data points are placed at the midpoint of the bins. The *inner* (*outer*) *error bars* indicate the statistical (combined statistical and systematic) uncertainties. The measurements are compared to predictions from MadGraph $+$ pythia 6, powheg $+$ pythia 6, powheg $+$ herwig 6, and mc@nlo $+$ herwig 6. The *lower part of each plot* shows the ratio of the predictions to data

Figure 7 presents the normalized differential cross sections for the dilepton channels: the transverse momentum $p_{\mathrm {T}} ^{\ell }$ and the pseudorapidity $\eta _{\ell }$ of the leptons, and the transverse momentum $p_{\mathrm {T}} ^{\ell ^{+}\ell ^{-}}$ and the invariant mass $m_{\ell ^{+}\ell ^{-}}$ of the lepton pair. The distributions in the transverse momentum of the b jets $p_{\mathrm {T}} ^{{\mathrm{b}}}$ and their pseudorapidity $\eta _{{\mathrm{b}}}$ are shown in Fig.  8, together with the transverse momentum $p_{\mathrm {T}} ^{{\mathrm{b}} \overline{{\mathrm{b}}} }$ and invariant mass $m_{{\mathrm{b}} \overline{{\mathrm{b}}} }$ of the b jet pair. Predictions from MadGraph$+$pythia 6, powheg$+$pythia 6, powheg$+$herwig 6, and mc@nlo$+$herwig 6 are also presented for comparison.

In general, none of the examined predictions provides an accurate description of data for all measured lepton and b jet distributions. A steeper $p_{\mathrm {T}}$ spectrum is observed in data for the lepton and the b jet distributions compared to the predictions in both decay channels, which is best described by powheg$+$herwig 6. The lepton $p_{\mathrm {T}}$ in data is above the predictions for $p_{\mathrm {T}} ^{\ell } < 40\,\text {GeV}$, while it is below for $p_{\mathrm {T}} ^{\ell } > 100\,\text {GeV}$. A similar behaviour is observed for $p_{\mathrm {T}} ^{\ell ^{+}\ell ^{-}}$, $p_{\mathrm {T}} ^{{\mathrm{b}}}$, and $p_{\mathrm {T}} ^{{\mathrm{b}} \overline{{\mathrm{b}}} }$. The $m_{\ell ^{+}\ell ^{-}}$ distribution in data is below all predictions for $m_{\ell ^{+}\ell ^{-}} > 30\,\text {GeV}$. Worse agreement is found for powheg$+$pythia 6. The $\eta$ distributions in data are described by the predictions within the experimental uncertainties. The $\eta _{{\mathrm{b}}}$ distributions are slightly less central in data than in the predictions, and are worse described by MadGraph$+$pythia 6. The remaining distributions are described by the predictions within experimental uncertainties.

### Top quark and ${\mathrm{t}}\overline{{\mathrm{t}}}$ differential cross sections

The normalized differential ${\mathrm{t}}\overline{{\mathrm{t}}}$ cross section as a function of the kinematic properties of the top quarks and the ${\mathrm{t}}\overline{{\mathrm{t}}}$ system is defined with respect to the top quarks or antiquarks before the decay (parton level) and after QCD radiation, and extrapolated to the full phase space using the MadGraph$+$pythia 6 prediction for the $\ell +$jets and dilepton channels.

**Fig. 9:**
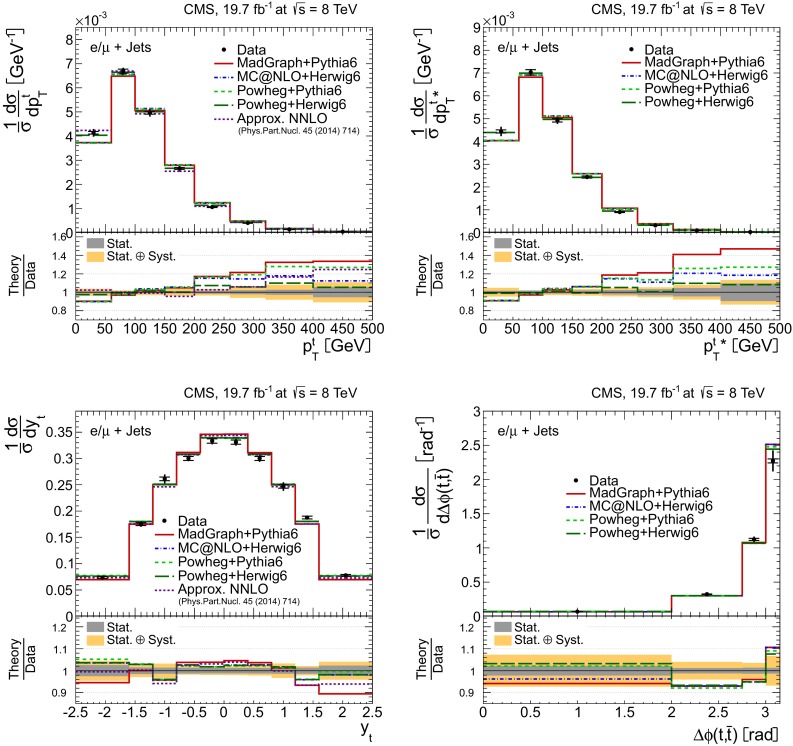
Normalized differential ${\mathrm{t}}\overline{{\mathrm{t}}}$ production cross section in the $\ell +$jets channels as a function of the $p_{\mathrm {T}} ^{{\mathrm{t}}}$ (*top left*), the ${\mathrm{t}}\overline{{\mathrm{t}}}$ rest frame $p_{\mathrm {T}} ^{{\mathrm{t}} *}$ (*top right*), and the rapidity $y_{{\mathrm{t}}}$ (*bottom left*) of the top quarks or antiquarks, and the difference in the azimuthal angle between the top quark and the antiquark $\Delta \phi (\text {t,}\bar{{\mathrm{t}}})$ (*bottom right*). The data points are placed at the midpoint of the bins. The *inner* (*outer*) *error bars* indicate the statistical (combined statistical and systematic) uncertainties. The measurements are compared to predictions from MadGraph $+$ pythia 6, powheg $+$ pythia 6, powheg $+$ herwig 6, mc@nlo $+$ herwig 6, and to approximate NNLO [16] calculations, when available. The *lower part of each plot* shows the ratio of the predictions to data

**Fig. 10:**
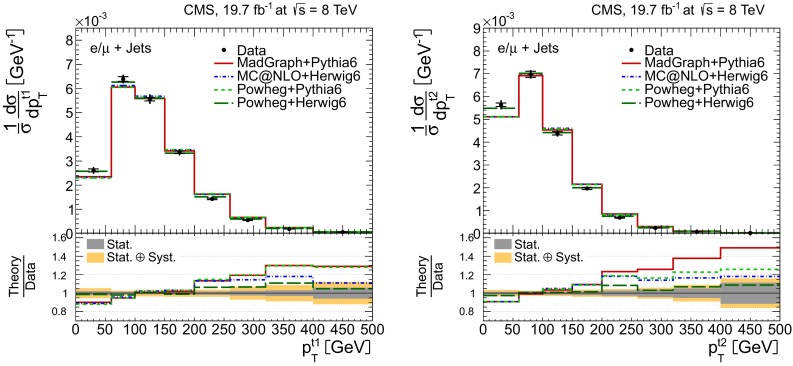
Normalized differential ${\mathrm{t}}\overline{{\mathrm{t}}}$ production cross section in the $\ell +$jets channels as a function of the $p_{\mathrm {T}}$ of the leading (*left*) and trailing (*right*) top quarks or antiquarks. The data points are placed at the midpoint of the bins. The *inner* (*outer*) *error bars* indicate the statistical (combined statistical and systematic) uncertainties. The measurements are compared to predictions from MadGraph $+$ pythia 6, powheg $+$ pythia 6, powheg $+$ herwig 6, and mc@nlo $+$ herwig 6. The *lower part of each plot* shows the ratio of the predictions to data

**Fig. 11:**
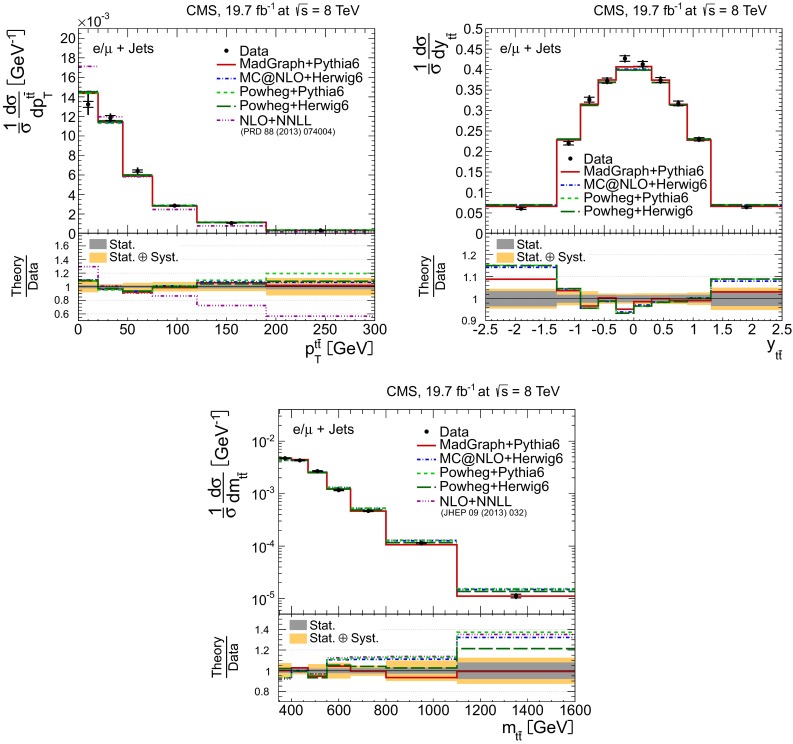
Normalized differential ${\mathrm{t}}\overline{{\mathrm{t}}}$ production cross section in the $\ell +$jets channels as a function of the $p_{\mathrm {T}} ^{{\mathrm{t}}\overline{{\mathrm{t}}}}$ (*top left*), $y_{{\mathrm{t}}\overline{{\mathrm{t}}}}$ (*top right*), and $m_{{\mathrm{t}}\overline{{\mathrm{t}}}}$ (*bottom*) of the ${\mathrm{t}}\overline{{\mathrm{t}}}$ system. The data points are placed at the midpoint of the bins. The *inner* (*outer*) *error bars* indicate the statistical (combined statistical and systematic) uncertainties. The measurements are compared to predictions from MadGraph $+$ pythia 6, powheg $+$ pythia 6, powheg $+$ herwig 6, mc@nlo $+$ herwig 6, and to NLO$+$NNLL [14, 15] calculations, when available. The *lower part of each plot* shows the ratio of the predictions to data

**Fig. 12:**
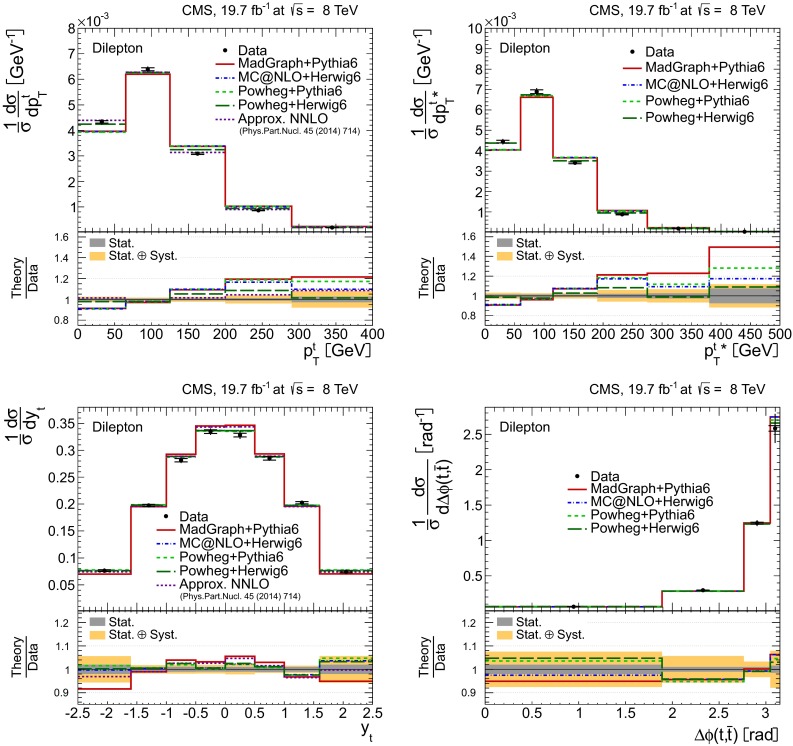
Normalized differential ${\mathrm{t}}\overline{{\mathrm{t}}}$ production cross section in the dilepton channels as a function of the $p_{\mathrm {T}} ^{{\mathrm{t}}}$ (*top left*), the ${\mathrm{t}}\overline{{\mathrm{t}}}$ rest frame $p_{\mathrm {T}} ^{{\mathrm{t}} *}$ (*top right*), and the rapidity $y_{{\mathrm{t}}}$ (*bottom left*) of the top quarks or antiquarks, and the difference in the azimuthal angle between the top quark and the antiquark $\Delta \phi (\text {t,}\bar{{\mathrm{t}}})$ (*bottom right*). The data points are placed at the midpoint of the bins. The *inner* (*outer*) *error bars* indicate the statistical (combined statistical and systematic) uncertainties. The measurements are compared to predictions from MadGraph $+$ pythia 6, powheg $+$ pythia 6, powheg $+$ herwig 6, mc@nlo $+$ herwig 6, and to approximate NNLO [16] calculations, when available. The *lower part of each plot* shows the ratio of the predictions to data

**Fig. 13:**
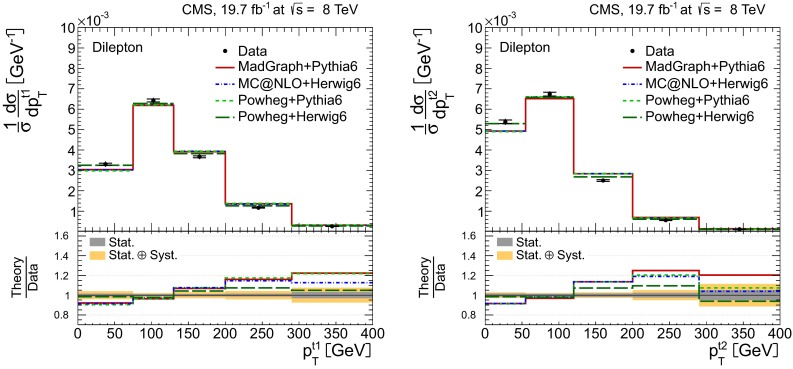
Normalized differential ${\mathrm{t}}\overline{{\mathrm{t}}}$ production cross section in the dilepton channels as a function of the $p_{\mathrm {T}}$ of the leading (*left*) and trailing (*right*) top quarks or antiquarks. The data points are placed at the midpoint of the bins. The *inner* (*outer*) *error bars* indicate the statistical (combined statistical and systematic) uncertainties. The measurements are compared to predictions from MadGraph $+$ pythia 6, powheg $+$ pythia 6, powheg $+$ herwig 6, and mc@nlo $+$ herwig 6. The *lower part of each plot* shows the ratio of the predictions to data

**Fig. 14:**
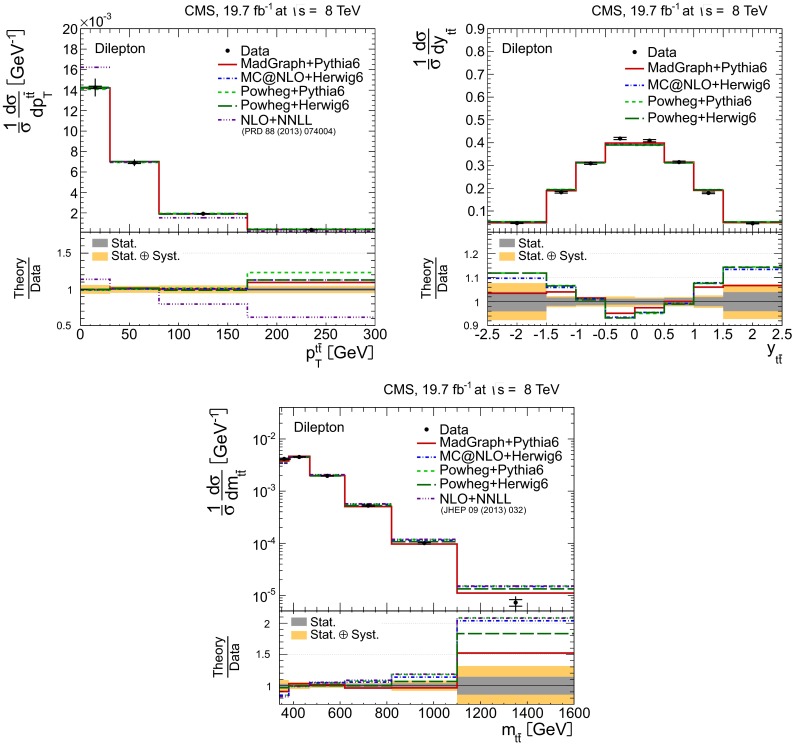
Normalized differential ${\mathrm{t}}\overline{{\mathrm{t}}}$ production cross section in the dilepton channels as a function of the $p_{\mathrm {T}} ^{{\mathrm{t}}\overline{{\mathrm{t}}}}$ (*top left*), $y_{{\mathrm{t}}\overline{{\mathrm{t}}}}$ (*top right*), and $m_{{\mathrm{t}}\overline{{\mathrm{t}}}}$ (*bottom*) of the ${\mathrm{t}}\overline{{\mathrm{t}}}$ system. The data points are placed at the midpoint of the bins. The *inner* (*outer*) *error bars* indicate the statistical (combined statistical and systematic) uncertainties. The measurements are compared to predictions from MadGraph $+$ pythia 6, powheg $+$ pythia 6, powheg $+$ herwig 6, mc@nlo $+$ herwig 6, and to NLO$+$NNLL [14, 15] calculations, when available. The *lower part of each plot* shows the ratio of the predictions to data

In Figs. 9, 10 and 11, the following distributions are presented for the $\ell +$jets channels: the transverse momentum $p_{\mathrm {T}} ^{{\mathrm{t}}}$ and the rapidity $y_{{\mathrm{t}}}$ of the top quarks or antiquarks, the transverse momentum $p_{\mathrm {T}} ^{{\mathrm{t}} *}$ of the top quarks or antiquarks in the ${\mathrm{t}}\overline{{\mathrm{t}}}$ rest frame, the difference in the azimuthal angle between the top quark and antiquark $\Delta \phi (\text {t,}\bar{{\mathrm{t}}})$, the transverse momentum of the leading ($p_{\mathrm {T}} ^{\text {t1}}$) and trailing ($p_{\mathrm {T}} ^{\text {t2}}$) top quark or antiquark, and the transverse momentum $p_{\mathrm {T}} ^{{\mathrm{t}}\overline{{\mathrm{t}}}}$, the rapidity $y_{{\mathrm{t}}\overline{{\mathrm{t}}}}$, and the invariant mass $m_{{\mathrm{t}}\overline{{\mathrm{t}}}}$ of the ${\mathrm{t}}\overline{{\mathrm{t}}}$ system. The data are compared to predictions from MadGraph$+$pythia 6, powheg$+$pythia 6, powheg$+$herwig 6, and mc@nlo$+$herwig 6. In addition, the approximate NNLO calculation [16] is also shown for the top quark $p_{\mathrm {T}}$ and rapidity results, while the $m_{{\mathrm{t}}\overline{{\mathrm{t}}}}$ and the $p_{\mathrm {T}} ^{{\mathrm{t}}\overline{{\mathrm{t}}}}$ distributions are compared to the NLO$+$NNLL predictions from Refs. [14, 15], respectively. Figures 12, 13 and 14 show the corresponding distributions in the dilepton channels. The lower panel in each plot also shows the ratio of each prediction relative to data.

In general, the powheg$+$herwig 6 prediction provides a good description of data for all measured distributions. The shape of the top quark $p_{\mathrm {T}}$ spectrum is softer in data than in the predictions from MadGraph$+$pythia 6, powheg$+$pythia 6, and mc@nlo$+$herwig 6 in both channels. The data lie above the predictions for $p_{\mathrm {T}} ^{{\mathrm{t}}} < 60$ (65)$\,\text {GeV}$ in the $\ell +$jets (dilepton) channels, while they lie below for $p_{\mathrm {T}} ^{{\mathrm{t}}} > 200\,\text {GeV}$. This effect was also observed at 7$\,\text {TeV}$  [5]. The disagreement between data and predictions in the tail of the distributions is also observed in a measurement by the ATLAS Collaboration [6]. In contrast, the prediction from powheg$+$herwig 6 and the approximate NNLO calculation provide a better description of the data, as they predict a slightly softer top quark $p_{\mathrm {T}}$ distribution than the three other simulations. The difference between the powheg$+$pythia 6 and powheg$+$herwig 6 distributions is attributed to different treatment of the hardest initial state radiation in pythia 6 and herwig 6. The same pattern is observed for $p_{\mathrm {T}} ^{{\mathrm{t}} *}$, indicating that the softer spectrum in data is not caused by the boost of the ${\mathrm{t}}\overline{{\mathrm{t}}}$ system. It is also present in the $p_{\mathrm {T}} ^{\text {t1}}$, and particularly, in the $p_{\mathrm {T}} ^{\text {t2}}$ distributions. For all these distributions, the powheg$+$herwig 6 prediction provides a better description of the data. The difference in the shape of the top quark $p_{\mathrm {T}}$ spectrum between data and simulation is observed consistently in the analyses using different event selection requirements or different pileup conditions. The $y_{t}$ distribution is found to be slightly less central in data than in the predictions, particularly in the case of MadGraph$+$pythia 6 and the approximate NNLO calculation, which are more central than the other predictions. On the contrary, $y_{{\mathrm{t}}\overline{{\mathrm{t}}}}$ is more central in data, and it is slightly better described by MadGraph$+$pythia 6. The $m_{{\mathrm{t}}\overline{{\mathrm{t}}}}$ distribution in data tends to be lower than the predictions for large $m_{{\mathrm{t}}\overline{{\mathrm{t}}}}$ values, and is better described by MadGraph$+$pythia 6 and powheg$+$herwig 6. The $p_{\mathrm {T}} ^{{\mathrm{t}}\overline{{\mathrm{t}}}}$ spectrum is well described by all the considered predictions, except for the NLO$+$NNLL calculation, which fails to describe the data for all $p_{\mathrm {T}} ^{{\mathrm{t}}\overline{{\mathrm{t}}}}$ values.

**Fig. 15:**
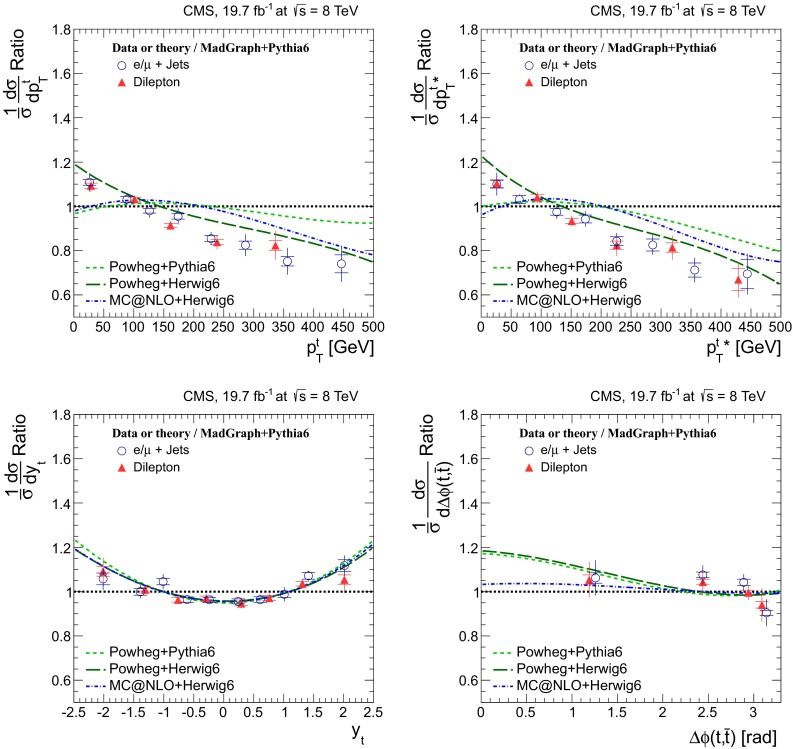
Comparison of normalized differential ${\mathrm{t}}\overline{{\mathrm{t}}}$ production cross section in the dilepton and $\ell +$jets channels as a function of the $p_{\mathrm {T}} ^{{\mathrm{t}}}$ (*top left*), the ${\mathrm{t}}\overline{{\mathrm{t}}}$ rest frame $p_{\mathrm {T}} ^{{\mathrm{t}} *}$ (*top righ*t), and the rapidity $y_{{\mathrm{t}}}$ (*bottom left*) of the top quarks or antiquarks, and the difference in the azimuthal angle between the top quark and the antiquark $\Delta \phi (\text {t,}\bar{{\mathrm{t}}})$ (*bottom righ*t). The measurements are presented relative to the MadGraph $+$ pythia 6 prediction. A horizontal bin-centre correction is applied to all data points (cf. Sect. 6.2). The *inner* (*outer*) *error bars* indicate the statistical (combined statistical and systematic) uncertainties. The predictions from powheg $+$ pythia 6, powheg $+$ herwig 6, and mc@nlo $+$ herwig 6, also presented relative to MadGraph $+$ pythia 6, are shown for comparison

**Fig. 16:**
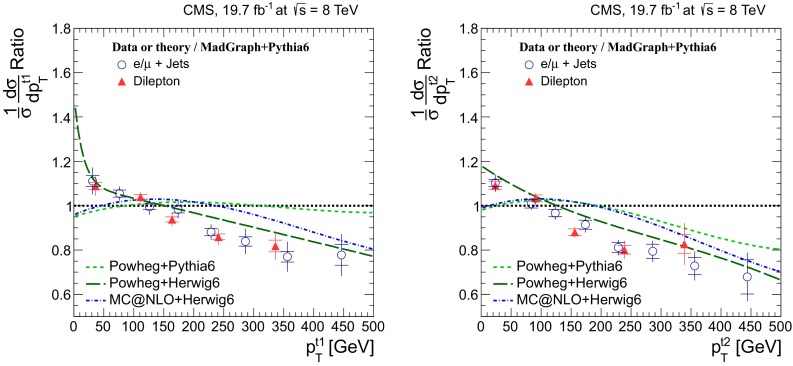
Comparison of normalized differential ${\mathrm{t}}\overline{{\mathrm{t}}}$ production cross section in the dilepton and $\ell +$jets channels as a function of the $p_{\mathrm {T}}$ of the leading (*left*) and trailing (*right*) top quarks or antiquarks. The measurements are presented relative to the MadGraph $+$ pythia 6 prediction. A *horizontal* bin-centre correction is applied to all data points (cf. Sect. 6.2). The *inner* (*outer*) *error bars* indicate the statistical (combined statistical and systematic) uncertainties. The predictions from powheg $+$ pythia 6, powheg $+$ herwig 6, and mc@nlo $+$ herwig 6, also presented relative to MadGraph $+$ pythia 6, are shown for comparison

**Fig. 17:**
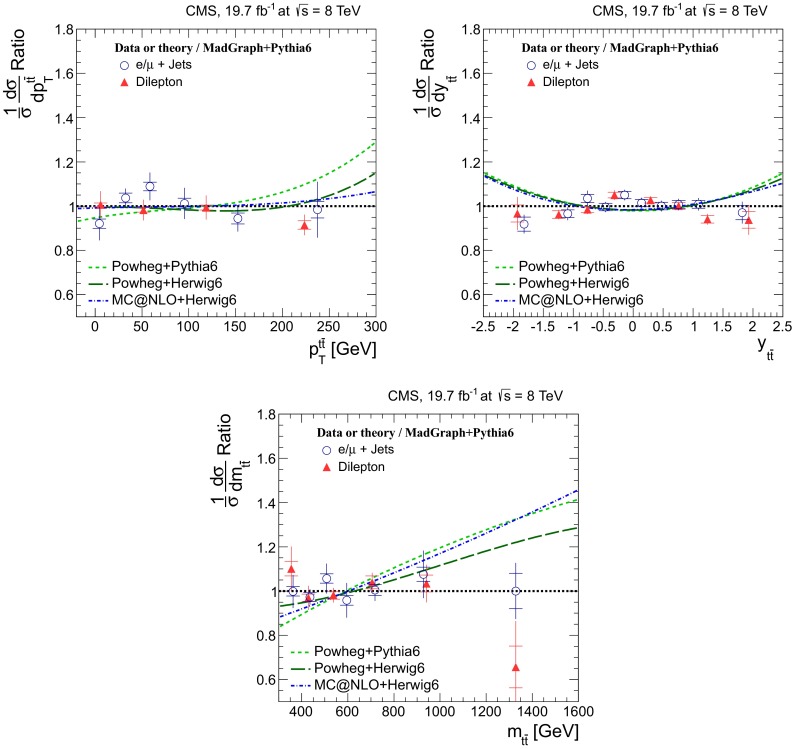
Comparison of normalized differential ${\mathrm{t}}\overline{{\mathrm{t}}}$ production cross section in the dilepton and $\ell +$jets channels as a function of the $p_{\mathrm {T}} ^{{\mathrm{t}}\overline{{\mathrm{t}}}}$ (*top left*), $y_{{\mathrm{t}}\overline{{\mathrm{t}}}}$ (*top right*), and $m_{{\mathrm{t}}\overline{{\mathrm{t}}}}$ (*bottom*) of the ${\mathrm{t}}\overline{{\mathrm{t}}}$ system. The measurements are presented relative to the MadGraph $+$ pythia 6 prediction. A *horizontal* bin-centre correction is applied to all data points (cf. Sect. 6.2). The *inner* (*outer*) *error bars* indicate the statistical (combined statistical and systematic) uncertainties. The predictions from powheg $+$ pythia 6, powheg $+$ herwig 6, and mc@nlo $+$ herwig 6, also presented relative to MadGraph $+$ pythia 6, are shown for comparison. For better visibility, data points with identical bin centres (cf. Supplemental Tables 6, 10) are shifted *horizontally* by a negligible amount

The results from the $\ell +$jets and dilepton channels are compared to each other in Figs. 15, 16 and 17. This is only feasible for the top quark and ${\mathrm{t}}\overline{{\mathrm{t}}}$ quantities, since they are measured in the same phase space (i.e. the full parton level phase space) for both channels. The results are presented relative to the MadGraph$+$pythia 6 prediction to highlight the level of agreement between data and the default ${\mathrm{t}}\overline{{\mathrm{t}}}$ simulation. To facilitate the comparison of measurements that are performed using different size and number of bins, a horizontal bin-centre correction is applied to all data points from both channels. In each bin, the measured data points are presented at the horizontal position in the bin where the predicted bin-averaged cross section equals the cross section of the unbinned MadGraph$+$pythia 6 calculation (cf. [51]), which is common for both channels. The data are also compared to the predictions from powheg$+$pythia 6, powheg$+$herwig 6, mc@nlo$+$herwig 6 relative to MadGraph$+$pythia 6. The results are consistent between the channels for all quantities, in particular, for all measurements related to the top quark $p_{\mathrm {T}}$ distribution. The softer spectrum in data relative to MadGraph$+$pythia 6 is clearly visible.

**Fig. 18:**
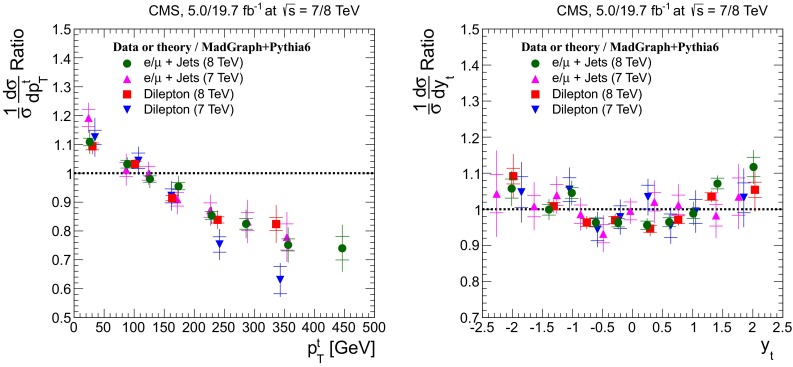
Comparison of normalized differential ${\mathrm{t}}\overline{{\mathrm{t}}}$ production cross section in the dilepton and $\ell +$jets channels at 7$\,\text {TeV}$  [5] and 8$\,\text {TeV}$, as a function of the $p_{\mathrm {T}} ^{{\mathrm{t}}}$ (*left*) and rapidity $y_{{\mathrm{t}}}$ (*right*) of the top quarks or antiquarks. The measurements are presented relative to the corresponding MadGraph $+$ pythia 6 predictions. A *horizontal* bin-centre correction is applied to all data points (cf. Sect. 6.2). The *inner* (*outer*) *error bars* indicate the statistical (combined statistical and systematic) uncertainties. For better visibility, data points with identical bin centres (cf. Supplemental Tables 6, 10) are shifted *horizontally* by a negligible amount

**Fig. 19:**
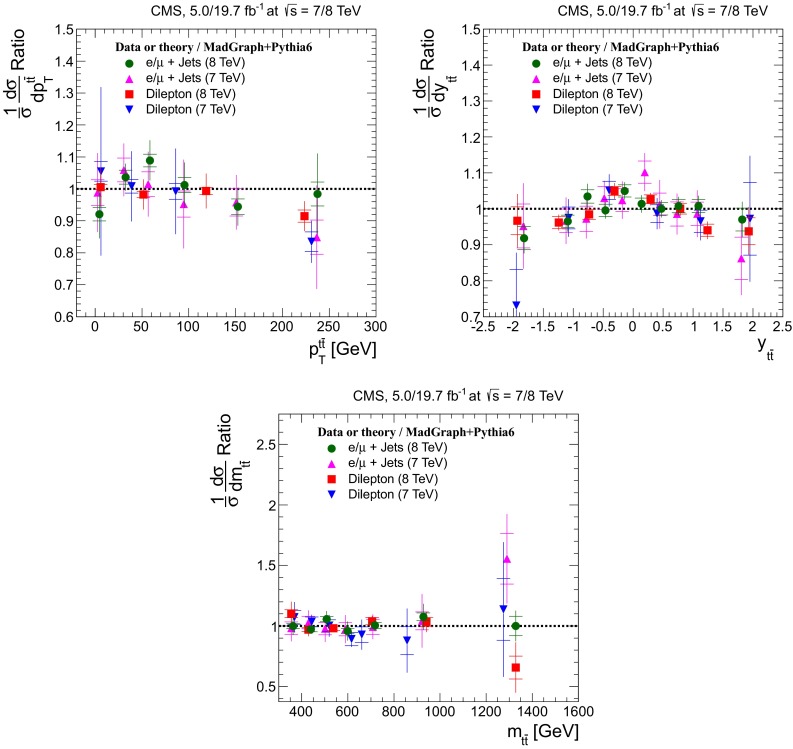
Comparison of normalized differential ${\mathrm{t}}\overline{{\mathrm{t}}}$ production cross section in the dilepton and $\ell +$jets channels at 7$\,\text {TeV}$  [5] and 8$\,\text {TeV}$, as a function of the $p_{\mathrm {T}} ^{{\mathrm{t}}\overline{{\mathrm{t}}}}$ (*top left*), $y_{{\mathrm{t}}\overline{{\mathrm{t}}}}$ (*top right*), and $m_{{\mathrm{t}}\overline{{\mathrm{t}}}}$ (*bottom*) of the ${\mathrm{t}}\overline{{\mathrm{t}}}$ system. The measurements are presented relative to the corresponding MadGraph $+$ pythia 6 predictions. A *horizontal* bin-centre correction is applied to all data points (cf. Sect. 6.2). The *inner* (*outer*) *error bars* indicate the statistical (combined statistical and systematic) uncertainties. For better visibility, data points with identical bin centres (cf. Supplemental Tables 9, 12) are shifted *horizontally* by a negligible amount

In addition, a comparison between results obtained at $\sqrt{s}=7$ [5] and 8$\,\text {TeV}$ is also performed for both the $\ell +$jets and dilepton channels, and presented in Figs. 18 and 19 for $p_{\mathrm {T}} ^{{\mathrm{t}}}$, $y_{{\mathrm{t}}}$, $p_{\mathrm {T}} ^{{\mathrm{t}}\overline{{\mathrm{t}}}}$, $y_{{\mathrm{t}}\overline{{\mathrm{t}}}}$, and $m_{{\mathrm{t}}\overline{{\mathrm{t}}}}$. Since the fiducial phase space definition for the normalized differential cross sections is also different for each value of $\sqrt{s}$, the comparison is again only possible for top quark and ${\mathrm{t}}\overline{{\mathrm{t}}}$ quantities. The measurements are presented relative to the corresponding default MadGraph$+$pythia 6 predictions at 7 and 8$\,\text {TeV}$. A horizontal bin-centre correction with respect to the MadGraph$+$pythia 6 predictions is applied to all data points from both channels and $\sqrt{s}$ values. The results are consistent between the channels for all quantities, both at 7 and 8$\,\text {TeV}$. The uncertainties in almost all bins of the distributions are reduced for the 8$\,\text {TeV}$ results relative to 7$\,\text {TeV}$, mainly due to the improvements discussed in Sect. 4.3. The softer $p_{\mathrm {T}} ^{{\mathrm{t}}}$ in data relative to MadGraph$+$pythia 6 is also visible at 7$\,\text {TeV}$.

## Summary

First measurements are presented of normalized differential ${\mathrm{t}}\overline{{\mathrm{t}}}$ production cross sections in pp collisions at $\sqrt{s}=8\,\text {TeV}$. The measurements are performed with the CMS detector in the $\ell +$jets ($\ell = \mathrm {e}\text { or }\mu$) and dilepton ($\mathrm {e}^+\mathrm {e}^-$, $\mu ^+ \mu ^-$, and $\mathrm {e}^\pm \mu ^{\mp }$) ${\mathrm{t}}\overline{{\mathrm{t}}}$ decay channels. The normalized ${\mathrm{t}}\overline{{\mathrm{t}}}$ cross section is measured as a function of the transverse momentum, rapidity, and invariant mass of the final-state leptons and b jets in the fiducial phase space, and the top quarks and ${\mathrm{t}}\overline{{\mathrm{t}}}$ system in the full phase space. The measurements in the different decay channels are in agreement with each other. In general, the data are in agreement with standard model predictions up to approximate NNLO precision. Among the examined predictions, powheg$+$herwig 6 provides the best overall description of the data. However, the $p_{\mathrm {T}}$ spectrum in data for leptons, jets, and top quarks is softer than expected, particularly for MadGraph$+$pythia 6, powheg$+$pythia 6, and mc@nlo$+$herwig 6. The calculation at approximate NNLO precision also provides a good description of the top quark $p_{\mathrm {T}}$ spectrum. The $m_{{\mathrm{t}}\overline{{\mathrm{t}}}}$ distribution in data tends to be lower than the predictions for large $m_{{\mathrm{t}}\overline{{\mathrm{t}}}}$ values. The $p_{\mathrm {T}} ^{{\mathrm{t}}\overline{{\mathrm{t}}}}$ spectrum is well described by all the considered predictions, except for the NLO$+$NNLL calculation, which fails to describe the data for all $p_{\mathrm {T}} ^{{\mathrm{t}}\overline{{\mathrm{t}}}}$ values. The results show the same behaviour as the corresponding CMS measurements at $\sqrt{s}=7\,\text {TeV}$.

## Electronic supplementary material

Supplementary material 1 (pdf 192 KB)
