# Supplementary material for: Measurement of the differential cross section for top quark pair production in pp collisions at √s = 8 TeV
Source: Eur Phys J C Part Fields. 2015 Nov 20;75:542. doi: 10.1140/epjc/s10052-015-3709-x (PMC4657759; doi:10.1140/epjc/s10052-015-3709-x)
Supplement: Supplementary file 1 — Supplementary material 1 (pdf 192 KB) [file 10052_2015_3709_MOESM1_ESM.pdf]

e-mail: cms-publication-committee-chair@cern.ch

Key words. CMS, physics, top quarks, differential cross sections

European Physical Journal C manuscript No.
(will be inserted by the editor)

Measurement of the differential cross section for top quark pair production in pp collisions at $\sqrt{s} = 8$ TeV

—Supplemental Material—

The CMS Collaboration

CERN

September 20, 2015

Abstract. The numerical results for the normalized $\mathrm{t\bar{t}}$ differential distributions are summarized. For each distribution, the result of the measurement, together with the bin range, as well as the statistical, systematic, and total uncertainties, are provided.

In Tables 1 to 12, the results for the normalized differential distributions are summarized. For each distribution, the result of the measurement, together with the bin range, as well as the statistical, systematic, and total uncertainties, are provided. For the top quark or antiquark and $\mathrm{t\bar{t}}$ quantities, the bin centres corrected according to the MadGraph+pythia6 prediction (cf. Section 6.2 (main document)) are also presented.

Table 1. Normalized differential $t\bar{t}$ cross section in the ℓ+jets channels as a function of the charged lepton transverse momentum (p_T^ℓ) and pseudorapidity (η_ℓ). The superscript 'ℓ' refers to both ℓ^+ and ℓ^-. The results are presented at particle level in the fiducial phase space. The statistical and systematic uncertainties are added in quadrature to yield the total uncertainty.

p_T^ℓ bin range [GeV]	$1/\sigma\, d\sigma/dp_T^\ell$ [GeV^{-1}]	Stat. [%]	Syst. [%]	Total [%]
$[30, 37)$	1.43×10^{-2}	2.2	2.4	3.2
$[37, 45)$	2.21×10^{-2}	1.4	2.8	3.1
$[45, 55)$	1.83×10^{-2}	1.4	1.7	2.3
$[55, 68)$	1.36×10^{-2}	1.5	1.4	2.1
$[68, 80)$	9.42×10^{-3}	2.0	1.5	2.5
$[80, 100)$	5.89×10^{-3}	1.8	1.2	2.2
$[100, 135)$	2.51×10^{-3}	2.1	6.3	6.7
$[135, 200)$	5.73×10^{-4}	3.3	9.5	10.1
η_ℓ bin range	$1/\sigma\, d\sigma/d\eta_\ell$	Stat. [%]	Syst. [%]	Total [%]
$[-2.1, -1.8)$	9.76×10^{-2}	4.3	4.3	6.0
$[-1.8, -1.5)$	1.37×10^{-1}	3.3	3.1	4.5
$[-1.5, -1.2)$	1.94×10^{-1}	2.7	2.4	3.6
$[-1.2, -0.9)$	2.39×10^{-1}	2.5	2.8	3.7
$[-0.9, -0.6)$	3.03×10^{-1}	2.0	2.3	3.0
$[-0.6, -0.3)$	3.41×10^{-1}	1.9	1.4	2.3
$[-0.3, 0.0)$	3.60×10^{-1}	1.9	1.4	2.4
$[0.0, 0.3)$	3.39×10^{-1}	2.0	2.0	2.8
$[0.3, 0.6)$	3.35×10^{-1}	1.9	3.0	3.6
$[0.6, 0.9)$	3.06×10^{-1}	1.9	4.2	4.6
$[0.9, 1.2)$	2.25×10^{-1}	2.2	2.2	3.2
$[1.2, 1.5)$	2.01×10^{-1}	2.6	2.2	3.4
$[1.5, 1.8)$	1.36×10^{-1}	3.3	2.5	4.2
$[1.8, 2.1)$	9.31×10^{-2}	4.2	5.6	7.0

Table 2. Normalized differential $\mathrm{t\bar{t}}$ cross section in the ℓ+jets channels as a function of the b jet transverse momentum ($p_{\mathrm{T}}^{\mathrm{b}}$) and pseudorapidity ($\eta_{\mathrm{b}}$), and the transverse momentum ($p_{\mathrm{T}}^{\mathrm{b\bar{b}}}$) and the invariant mass ($m_{\mathrm{b\bar{b}}}$) of the $\mathrm{b\bar{b}}$ system. The superscript 'b' refers to both b and $\mathrm{\bar{b}}$ jets. The results are presented at particle level in the fiducial phase space. The statistical and systematic uncertainties are added in quadrature to yield the total uncertainty.

$p_{\mathrm{T}}^{\mathrm{b}}$ bin range [GeV]	$1/\sigma\, \mathrm{d}\sigma/\mathrm{d}p_{\mathrm{T}}^{\mathrm{b}}$ [GeV^{-1}]	Stat. [%]	Syst. [%]	Total [%]
[30, 48)	1.40×10^{-2}	1.2	7.0	7.1
[48, 75)	1.24×10^{-2}	0.9	1.8	2.0
[75, 180)	3.67×10^{-3}	0.7	4.9	5.0
[180, 400)	1.34×10^{-4}	3.3	12.4	12.9
η_{b} bin range	$1/\sigma\, \mathrm{d}\sigma/\mathrm{d}\eta_{\mathrm{b}}$	Stat. [%]	Syst. [%]	Total [%]
[−2.4, −1.5)	1.01×10^{-1}	1.7	2.9	3.3
[−1.5, −1.0)	2.08×10^{-1}	1.3	1.5	2.0
[−1.0, −0.5)	2.77×10^{-1}	1.1	1.2	1.7
[−0.5, 0.0)	3.23×10^{-1}	1.1	2.0	2.2
[0.0, 0.5)	3.28×10^{-1}	1.1	1.9	2.1
[0.5, 1.0)	2.89×10^{-1}	1.1	1.0	1.5
[1.0, 1.5)	2.09×10^{-1}	1.4	2.2	2.6
[1.5, 2.4)	1.03×10^{-1}	1.6	2.8	3.2
$p_{\mathrm{T}}^{\mathrm{b\bar{b}}}$ bin range [GeV]	$1/\sigma\, \mathrm{d}\sigma/\mathrm{d}p_{\mathrm{T}}^{\mathrm{b\bar{b}}}$ [GeV^{-1}]	Stat. [%]	Syst. [%]	Total [%]
[0, 35)	3.41×10^{-3}	2.4	4.1	4.7
[35, 75)	6.55×10^{-3}	1.7	3.0	3.5
[75, 115)	8.51×10^{-3}	1.5	3.0	3.4
[115, 155)	5.04×10^{-3}	2.0	6.7	7.0
[155, 280)	5.79×10^{-4}	3.8	11.3	11.9
[280, 500)	0.17×10^{-4}	14.1	23.3	27.2
$m_{\mathrm{b\bar{b}}}$ bin range [GeV]	$1/\sigma\, \mathrm{d}\sigma/\mathrm{d}m_{\mathrm{b\bar{b}}}$ [GeV^{-1}]	Stat. [%]	Syst. [%]	Total [%]
[0, 85)	2.00×10^{-3}	1.9	7.6	7.9
[85, 135)	5.79×10^{-3}	1.7	3.9	4.3
[135, 190)	4.44×10^{-3}	2.0	3.0	3.6
[190, 255)	2.37×10^{-3}	2.5	4.6	5.3
[255, 325)	1.13×10^{-3}	3.7	7.6	8.5
[325, 415)	4.50×10^{-4}	4.9	8.6	10.0
[415, 505)	1.57×10^{-4}	8.8	10.9	14.0
[505, 630)	0.47×10^{-4}	12.6	28.4	31.1
[630, 800)	0.12×10^{-4}	21.9	15.4	26.8

Table 3. Normalized differential $t\bar{t}$ cross section in the dilepton channels as a function of the charged lepton transverse momentum (p_T^ℓ) and pseudorapidity (η_ℓ). The superscript 'ℓ' refers to both ℓ^+ and ℓ^-. The results are presented at particle level in the fiducial phase space. The statistical and systematic uncertainties are added in quadrature to yield the total uncertainty.

p_T^ℓ bin range [GeV]	$1/\sigma\, d\sigma/dp_T^\ell$ [GeV^{-1}]	Stat. [%]	Syst. [%]	Total [%]
[20, 40)	1.93×10^{-2}	0.5	1.6	1.7
[40, 70)	1.25×10^{-2}	0.5	0.7	0.9
[70, 120)	3.79×10^{-3}	0.8	1.8	2.0
[120, 180)	6.51×10^{-4}	2.0	7.2	7.4
[180, 400)	3.89×10^{-5}	4.6	12.0	12.9
η_ℓ bin range	$1/\sigma\, d\sigma/d\eta_\ell$	Stat. [%]	Syst. [%]	Total [%]
[−2.4, −2.1)	6.04×10^{-2}	3.0	2.7	4.1
[−2.1, −1.8)	9.49×10^{-2}	2.2	2.9	3.7
[−1.8, −1.5)	1.40×10^{-1}	1.9	1.3	2.3
[−1.5, −1.2)	1.98×10^{-1}	1.5	1.5	2.2
[−1.2, −0.9)	2.43×10^{-1}	1.4	0.6	1.5
[−0.9, −0.6)	2.86×10^{-1}	1.2	2.3	2.6
[−0.6, −0.3)	3.22×10^{-1}	1.2	1.0	1.5
[−0.3, 0.0)	3.36×10^{-1}	1.2	0.7	1.4
[0.0, 0.3)	3.17×10^{-1}	1.2	1.2	1.7
[0.3, 0.6)	3.23×10^{-1}	1.2	1.2	1.7
[0.6, 0.9)	2.90×10^{-1}	1.3	1.1	1.7
[0.9, 1.2)	2.43×10^{-1}	1.4	1.0	1.7
[1.2, 1.5)	1.97×10^{-1}	1.6	1.2	2.0
[1.5, 1.8)	1.30×10^{-1}	2.1	2.6	3.4
[1.8, 2.1)	9.70×10^{-2}	2.3	3.1	3.8
[2.1, 2.4)	5.38×10^{-2}	3.5	5.2	6.3

Table 4. Normalized differential $t\bar{t}$ cross section in the dilepton channels as a function of the transverse momentum ($p_T^{\ell\ell}$) and the invariant mass ($m_{\ell\ell}$) of the dilepton pair. The superscript 'ℓ' refers to both ℓ^+ and ℓ^-. The results are presented at particle level in the fiducial phase space. The statistical and systematic uncertainties are added in quadrature to yield the total uncertainty.

$p_T^{\ell\ell}$ bin range [GeV]	$1/\sigma\, d\sigma/dp_T^{\ell\ell}$ [GeV^{-1}]	stat. [%]	Syst. [%]	Total [%]
[0, 10)	1.84×10^{-3}	3.7	3.2	4.9
[10, 20)	4.82×10^{-3}	2.0	2.8	3.5
[20, 40)	7.74×10^{-3}	1.2	2.8	3.0
[40, 60)	1.10×10^{-2}	1.0	2.2	2.5
[60, 100)	9.70×10^{-3}	0.7	0.9	1.2
[100, 150)	2.87×10^{-3}	1.3	5.0	5.2
[150, 400)	1.03×10^{-4}	3.4	7.0	7.8
$m_{\ell\ell}$ bin range [GeV]	$1/\sigma\, d\sigma/dm_{\ell\ell}$ [GeV^{-1}]	Stat. [%]	Syst. [%]	Total [%]
[20, 30)	3.74×10^{-3}	2.6	2.3	3.5
[30, 50)	5.28×10^{-3}	1.5	2.1	2.6
[50, 76)	7.61×10^{-3}	1.0	1.6	1.9
[76, 106)	7.19×10^{-3}	1.0	2.1	2.4
[106, 130)	5.41×10^{-3}	1.3	1.4	1.9
[130, 170)	3.30×10^{-3}	1.4	1.7	2.2
[170, 260)	1.23×10^{-3}	1.5	3.4	3.7
[260, 400)	2.29×10^{-4}	2.7	6.9	7.4

Table 5. Normalized differential $\mathrm{t\bar{t}}$ cross section in the dilepton channels as a function of the b jet transverse momentum ($p_{\mathrm{T}}^{\mathrm{b}}$) and pseudorapidity ($\eta_{\mathrm{b}}$), and the transverse momentum ($p_{\mathrm{T}}^{\mathrm{b\bar{b}}}$) and the invariant mass ($m_{\mathrm{b\bar{b}}}$) of the $\mathrm{b\bar{b}}$ system. The superscript 'b' refers to both b and $\bar{\mathrm{b}}$ jets. The results are presented at particle level in the fiducial phase space. The statistical and systematic uncertainties are added in quadrature to yield the total uncertainty.

$p_{\mathrm{T}}^{\mathrm{b}}$ bin range [GeV]	$1/\sigma\, \mathrm{d}\sigma/\mathrm{d}p_{\mathrm{T}}^{\mathrm{b}}$ [GeV^{-1}]	Stat. [%]	Syst. [%]	Total [%]
[30, 50)	1.16×10^{-2}	1.2	7.7	7.8
[50, 80)	1.23×10^{-2}	1.2	3.8	4.0
[80, 130)	5.94×10^{-3}	1.4	3.4	3.7
[130, 210)	1.11×10^{-3}	2.2	5.1	5.5
[210, 400)	6.88×10^{-5}	6.8	13.5	15.1
η_{b} bin range	$1/\sigma\, \mathrm{d}\sigma/\mathrm{d}\eta_{\mathrm{b}}$	Stat. [%]	Syst. [%]	Total [%]
[−2.4, −1.5)	1.08×10^{-1}	2.3	6.9	7.3
[−1.5, −1.0)	2.16×10^{-1}	2.0	1.8	2.7
[−1.0, −0.5)	2.74×10^{-1}	1.9	3.2	3.7
[−0.5, 0.0)	3.01×10^{-1}	1.9	3.2	3.7
[0.0, 0.5)	3.20×10^{-1}	1.8	2.6	3.2
[0.5, 1.0)	2.78×10^{-1}	1.9	2.6	3.2
[1.0, 1.5)	2.19×10^{-1}	2.0	1.9	2.7
[1.5, 2.4)	1.09×10^{-1}	2.4	5.8	6.2
$p_{\mathrm{T}}^{\mathrm{b\bar{b}}}$ bin range [GeV]	$1/\sigma\, \mathrm{d}\sigma/\mathrm{d}p_{\mathrm{T}}^{\mathrm{b\bar{b}}}$ [GeV^{-1}]	Stat. [%]	Syst. [%]	Total [%]
[0, 30)	3.49×10^{-3}	2.3	3.6	4.3
[30, 60)	6.50×10^{-3}	1.5	2.9	3.3
[60, 100)	8.07×10^{-3}	1.2	2.1	2.4
[100, 180)	4.27×10^{-3}	1.0	3.2	3.4
[180, 400)	1.54×10^{-4}	4.4	9.8	10.7
$m_{\mathrm{b\bar{b}}}$ bin range [GeV]	$1/\sigma\, \mathrm{d}\sigma/\mathrm{d}m_{\mathrm{b\bar{b}}}$ [GeV^{-1}]	Stat. [%]	Syst. [%]	Total [%]
[0, 60)	8.48×10^{-4}	1.9	3.9	4.3
[60, 120)	4.57×10^{-3}	0.9	2.7	2.8
[120, 240)	3.97×10^{-3}	0.6	1.0	1.1
[240, 600)	5.35×10^{-4}	1.3	4.6	4.8

Table 6. Normalized differential $\mathrm{t\bar{t}}$ cross section in the ℓ+jets channels as a function of top quark or antiquark observables: the transverse momentum (p_T^t) and the transverse momentum in the $\mathrm{t\bar{t}}$ rest frame (p_T^{t*}) of the top quarks or antiquarks. The horizontally-corrected bin centres according to the MadGraph+pythia6 prediction (cf. Section 6.2 (main document)) are also provided. The results are presented at parton level in the full phase space. The statistical and systematic uncertainties are added in quadrature to yield the total uncertainty.

p_T^t bin range [GeV]	Bin centre [GeV]	$1/\sigma\, d\sigma/dp_T^t$ [GeV^{-1}]	Stat. [%]	Syst. [%]	Total [%]
[0, 60)	26.25	4.14×10^{-3}	1.2	3.6	3.8
[60, 100)	88.75	6.69×10^{-3}	1.3	1.7	2.1
[100, 150)	126.25	4.96×10^{-3}	1.1	3.0	3.2
[150, 200)	173.75	2.66×10^{-3}	1.3	3.5	3.7
[200, 260)	228.75	1.06×10^{-3}	1.6	3.2	3.6
[260, 320)	286.25	3.99×10^{-4}	2.2	5.6	6.0
[320, 400)	356.25	1.30×10^{-4}	2.8	7.6	8.1
[400, 500)	446.25	0.37×10^{-4}	5.5	9.5	10.9
p_T^{t*} bin range [GeV]	Bin centre [GeV]	$1/\sigma\, d\sigma/dp_T^{t*}$ [GeV^{-1}]	Stat. [%]	Syst. [%]	Total [%]
[0, 60)	26.25	4.44×10^{-3}	1.6	4.4	4.7
[60, 100)	63.75	7.03×10^{-3}	1.7	1.4	2.2
[100, 150)	126.25	4.93×10^{-3}	1.5	2.9	3.2
[150, 200)	173.75	2.44×10^{-3}	1.9	3.2	3.7
[200, 260)	226.25	9.00×10^{-4}	2.4	3.8	4.4
[260, 320)	286.25	3.21×10^{-4}	3.3	4.2	5.4
[320, 400)	356.25	0.94×10^{-4}	4.5	6.3	7.8
[400, 500)	443.75	0.25×10^{-4}	9.6	9.3	13.4

Table 7. Normalized differential $\mathrm{t\bar{t}}$ cross section in the ℓ+jets channels as a function of top quark or antiquark observables: the rapidity (y_t) of the top quarks or antiquarks, and the difference in the azimuthal angle between the top quark and antiquark ($\Delta\phi(\mathrm{t,\bar{t}})$). The horizontally-corrected bin centres according to the MadGraph+pythia6 prediction (cf. Section 6.2 (main document)) are also provided. The results are presented at parton level in the full phase space. The statistical and systematic uncertainties are added in quadrature to yield the total uncertainty.

y_t bin range	Bin centre	$1/\sigma\, d\sigma/dy_t$	Stat. [%]	Syst. [%]	Total [%]
[−2.5, −1.6)	−2.01	7.36×10^{-2}	2.5	3.8	4.5
[−1.6, −1.2)	−1.39	1.75×10^{-1}	1.5	2.4	2.9
[−1.2, −0.8)	−1.01	2.61×10^{-1}	1.4	3.0	3.3
[−0.8, −0.4)	−0.61	3.00×10^{-1}	1.3	1.3	1.8
[−0.4, 0.0)	−0.24	3.33×10^{-1}	1.2	1.5	1.9
[0.0, 0.4)	0.24	3.31×10^{-1}	1.2	1.6	2.0
[0.4, 0.8)	0.61	3.00×10^{-1}	1.3	1.8	2.2
[0.8, 1.2)	1.01	2.47×10^{-1}	1.4	3.0	3.3
[1.2, 1.6)	1.41	1.88×10^{-1}	1.4	1.6	2.1
[1.6, 2.5)	2.01	7.77×10^{-2}	2.4	3.4	4.1
$\Delta\phi(\mathrm{t,\bar{t}})$ bin range [rad]	Bin centre [rad]	$1/\sigma\, d\sigma/d\Delta\phi(\mathrm{t,\bar{t}})$ [rad^{-1}]	Stat. [%]	Syst. [%]	Total [%]
[0.00, 2.00)	1.26	6.83×10^{-2}	2.2	7.0	7.3
[2.00, 2.75)	2.44	3.22×10^{-1}	1.2	3.8	4.0
[2.75, 3.00)	2.89	1.13	1.4	3.2	3.5
[3.00, 3.15)	3.14	2.27	1.3	6.6	6.8

Table 8. Normalized differential $\mathrm{t\bar{t}}$ cross section in the ℓ+jets channels as a function of the p_{T} of the leading ($p_{\mathrm{T}}^{\mathrm{t}1}$) and trailing ($p_{\mathrm{T}}^{\mathrm{t}2}$) top quarks or antiquarks. The horizontally-corrected bin centres according to the MadGraph+pythia6 prediction (cf. Section 6.2 (main document)) are also provided. The results are presented at parton level in the full phase space. The statistical and systematic uncertainties are added in quadrature to yield the total uncertainty.

$p_{\mathrm{T}}^{\mathrm{t}1}$ bin range [GeV]	Bin centre [GeV]	$1/\sigma\, \mathrm{d}\sigma/\mathrm{d}p_{\mathrm{T}}^{\mathrm{t}1}$ [GeV^{-1}]	Stat. [%]	Syst. [%]	Total [%]
[0, 60)	31.25	2.61×10^{-3}	2.2	5.0	5.4
[60, 100)	76.25	6.39×10^{-3}	1.5	2.3	2.8
[100, 150)	126.25	5.56×10^{-3}	1.3	3.3	3.6
[150, 200)	173.75	3.36×10^{-3}	1.6	3.6	3.9
[200, 260)	228.75	1.43×10^{-3}	1.9	3.4	3.9
[260, 320)	286.25	5.56×10^{-4}	2.5	6.3	6.8
[320, 400)	356.25	1.87×10^{-4}	3.1	8.2	8.8
[400, 500)	446.25	0.56×10^{-4}	6.0	10.5	12.1
$p_{\mathrm{T}}^{\mathrm{t}2}$ bin range [GeV]	Bin centre [GeV]	$1/\sigma\, \mathrm{d}\sigma/\mathrm{d}p_{\mathrm{T}}^{\mathrm{t}2}$ [GeV^{-1}]	Stat. [%]	Syst. [%]	Total [%]
[0, 60)	23.75	5.64×10^{-3}	1.4	3.2	3.5
[60, 100)	83.75	6.98×10^{-3}	1.9	1.8	2.6
[100, 150)	123.75	4.39×10^{-3}	1.7	2.6	3.1
[150, 200)	173.75	1.97×10^{-3}	2.2	3.2	3.9
[200, 260)	228.75	6.93×10^{-4}	2.8	3.3	4.3
[260, 320)	286.25	2.43×10^{-4}	4.0	4.5	6.0
[320, 400)	356.25	0.75×10^{-4}	5.3	7.3	9.0
[400, 500)	443.75	0.19×10^{-4}	11.4	11.1	16.0

Table 9. Normalized differential $t\bar{t}$ cross section in the ℓ+jets channels as a function of top quark pair observables: the transverse momentum ($p_T^{t\bar{t}}$), the rapidity ($y_{t\bar{t}}$) and the invariant mass ($m_{t\bar{t}}$) of the $t\bar{t}$ system. The horizontally-corrected bin centres according to the MadGraph+pythia6 prediction (cf. Section 6.2 (main document)) are also provided. The results are presented at parton level in the full phase space. The statistical and systematic uncertainties are added in quadrature to yield the total uncertainty.

$p_T^{t\bar{t}}$ bin range [GeV]	Bin centre [GeV]	$1/\sigma\, d\sigma/dp_T^{t\bar{t}}$ [GeV^{-1}]	Stat. [%]	Syst. [%]	Total [%]
[0, 20)	4.50	1.32×10^{-2}	2.3	7.9	8.2
[20, 45)	32.50	1.18×10^{-2}	2.1	3.4	3.9
[45, 75)	58.50	6.40×10^{-3}	1.8	5.5	5.8
[75, 120)	95.50	2.84×10^{-3}	2.3	6.6	7.0
[120, 190)	152.50	1.07×10^{-3}	2.6	5.7	6.2
[190, 300)	237.50	3.06×10^{-4}	3.8	12.2	12.8
$y_{t\bar{t}}$ bin range	Bin centre	$1/\sigma\, d\sigma/dy_{t\bar{t}}$	Stat. [%]	Syst. [%]	Total [%]
[−2.5, −1.3)	−1.82	6.07×10^{-2}	3.5	2.9	4.5
[−1.3, −0.9)	−1.09	2.20×10^{-1}	1.8	2.3	3.0
[−0.9, −0.6)	−0.76	3.27×10^{-1}	1.8	2.9	3.4
[−0.6, −0.3)	−0.46	3.73×10^{-1}	1.7	1.6	2.3
[−0.3, 0.0)	−0.14	4.27×10^{-1}	1.6	1.6	2.2
[0.0, 0.3)	0.14	4.13×10^{-1}	1.6	1.9	2.4
[0.3, 0.6)	0.47	3.74×10^{-1}	1.7	1.7	2.4
[0.6, 0.9)	0.76	3.17×10^{-1}	1.8	1.7	2.5
[0.9, 1.3)	1.09	2.30×10^{-1}	1.8	2.2	2.8
[1.3, 2.5)	1.82	6.41×10^{-2}	3.3	3.8	5.1
$m_{t\bar{t}}$ bin range [GeV]	Bin centre [GeV]	$1/\sigma\, d\sigma/dm_{t\bar{t}}$ [GeV^{-1}]	Stat. [%]	Syst. [%]	Total [%]
[345, 400)	362.5 0	4.69×10^{-3}	2.1	7.1	7.5
[400, 470)	435.50	4.30×10^{-3}	2.1	2.9	3.6
[470, 550)	508.50	2.67×10^{-3}	2.0	6.1	6.4
[550, 650)	595.50	1.17×10^{-3}	2.3	7.3	7.7
[650, 800)	717.50	4.66×10^{-4}	2.5	4.2	4.9
[800, 1100)	927.50	1.14×10^{-4}	3.0	9.5	10.0
[1100, 1600)	1328.50	0.11×10^{-4}	8.0	9.8	12.7

Table 10. Normalized differential $\mathrm{t\bar{t}}$ cross section in the dilepton channels as a function of top quark or antiquark observables: the transverse momentum ($p_{\mathrm{T}}^{\mathrm{t}}$), the transverse momentum in the $\mathrm{t\bar{t}}$ rest frame ($p_{\mathrm{T}}^{\mathrm{t}*}$), and the rapidity ($y_{\mathrm{t}}$) of the top quarks or antiquarks, and the difference in the azimuthal angle between the top quark and antiquark ($\Delta\phi(\mathrm{t,\bar{t}})$). The horizontally-corrected bin centres according to the MadGraph+pythia6 prediction (cf. Section 6.2 (main document)) are also provided. The results are presented at parton level in the full phase space. The statistical and systematic uncertainties are added in quadrature to yield the total uncertainty.

$p_{\mathrm{T}}^{\mathrm{t}}$ bin range [GeV]	Bin centre [GeV]	$1/\sigma\, \mathrm{d}\sigma/\mathrm{d}p_{\mathrm{T}}^{\mathrm{t}}$ [GeV^{-1}]	Stat. [%]	Syst. [%]	Total [%]
$[0, 65)$	28.75	4.33×10^{-3}	1.1	2.3	2.5
$[65, 125)$	101.25	6.40×10^{-3}	0.9	1.4	1.7
$[125, 200)$	161.25	3.08×10^{-3}	1.0	2.1	2.3
$[200, 290)$	238.75	8.62×10^{-4}	1.3	3.6	3.8
$[290, 400)$	336.25	1.88×10^{-4}	2.7	7.4	7.9
$p_{\mathrm{T}}^{\mathrm{t}*}$ bin range [GeV]	Bin centre [GeV]	$1/\sigma\, \mathrm{d}\sigma/\mathrm{d}p_{\mathrm{T}}^{\mathrm{t}*}$ [GeV^{-1}]	Stat. [%]	Syst. [%]	Total [%]
$[0, 60)$	26.25	4.45×10^{-3}	1.5	2.7	3.1
$[60, 115)$	93.75	6.89×10^{-3}	1.4	2.1	2.5
$[115, 190)$	151.25	3.41×10^{-3}	1.3	2.2	2.6
$[190, 275)$	226.25	8.78×10^{-4}	2.2	5.5	5.9
$[275, 380)$	318.75	1.87×10^{-4}	2.6	6.2	6.7
$[380, 500)$	428.75	2.91×10^{-5}	7.5	9.1	11.8
y_{t} bin range	Bin centre	$1/\sigma\, \mathrm{d}\sigma/\mathrm{d}y_{\mathrm{t}}$	Stat. [%]	Syst. [%]	Total [%]
$[-2.5, -1.6)$	-2.01	7.63×10^{-2}	2.0	5.3	5.6
$[-1.6, -1.0)$	-1.31	1.97×10^{-1}	1.0	1.5	1.8
$[-1.0, -0.5)$	-0.76	2.82×10^{-1}	1.1	1.5	1.9
$[-0.5, 0.0)$	-0.29	3.35×10^{-1}	1.0	1.5	1.8
$[0.0, 0.5)$	0.29	3.28×10^{-1}	1.0	1.9	2.2
$[0.5, 1.0)$	0.76	2.85×10^{-1}	1.1	1.1	1.5
$[1.0, 1.6)$	1.31	2.03×10^{-1}	1.1	1.1	1.5
$[1.6, 2.5)$	2.01	7.40×10^{-2}	2.0	5.3	5.7
$\Delta\phi(\mathrm{t,\bar{t}})$ bin range [rad]	Bin centre [rad]	$1/\sigma\, \mathrm{d}\sigma/\mathrm{d}\Delta\phi(\mathrm{t,\bar{t}})$ [rad^{-1}]	Stat. [%]	Syst. [%]	Total [%]
$[0, 1.89)$	1.19	6.40×10^{-2}	2.0	7.3	7.6
$[1.89, 2.77)$	2.44	2.96×10^{-1}	1.0	5.6	5.7
$[2.77, 3.04)$	2.94	1.24	1.1	3.2	3.3
$[3.04, 3.15)$	3.09	2.58	1.5	7.8	8.0

Table 11. Normalized differential $\mathrm{t\bar{t}}$ cross section in the dilepton channels as a function of the p_{T} of the leading ($p_{\mathrm{T}}^{\mathrm{t1}}$) and trailing ($p_{\mathrm{T}}^{\mathrm{t2}}$) top quarks or antiquarks. The results are presented at parton level in the full phase space. The statistical and systematic uncertainties are added in quadrature to yield the total uncertainty.

$p_{\mathrm{T}}^{\mathrm{t1}}$ bin range [GeV]	Bin centre [GeV]	$1/\sigma\, \mathrm{d}\sigma/\mathrm{d}p_{\mathrm{T}}^{\mathrm{t1}}$ [GeV^{-1}]	Stat. [%]	Syst. [%]	Total [%]
$[0, 75)$	36.25	3.31×10^{-3}	1.5	3.7	4.0
$[75, 130)$	111.25	6.43×10^{-3}	1.1	1.8	2.1
$[130, 200)$	163.75	3.67×10^{-3}	1.2	2.9	3.2
$[200, 290)$	241.25	1.17×10^{-3}	1.5	3.9	4.2
$[290, 400)$	336.25	2.61×10^{-4}	3.2	6.9	7.6
$p_{\mathrm{T}}^{\mathrm{t2}}$ bin range [GeV]	Bin centre [GeV]	$1/\sigma\, \mathrm{d}\sigma/\mathrm{d}p_{\mathrm{T}}^{\mathrm{t2}}$ [GeV^{-1}]	Stat. [%]	Syst. [%]	Total [%]
$[0, 55)$	23.7 5	5.38×10^{-3}	1.7	2.2	2.8
$[55, 120)$	91.25	6.74×10^{-3}	1.4	1.6	2.1
$[120, 200)$	156.25	2.50×10^{-3}	1.7	2.1	2.7
$[200, 290)$	238.75	5.58×10^{-4}	2.4	4.6	5.2
$[290, 400)$	338.75	1.14×10^{-4}	5.1	10.2	11.4

Table 12. Normalized differential $\mathrm{t\bar{t}}$ cross section in the dilepton channels as a function of top quark pair observables: the transverse momentum ($p_{\mathrm{T}}^{\mathrm{t\bar{t}}}$), the rapidity ($y_{\mathrm{t\bar{t}}}$) and the invariant mass ($m_{\mathrm{t\bar{t}}}$) of the $\mathrm{t\bar{t}}$ system. The results are presented at parton level in the full phase space. The statistical and systematic uncertainties are added in quadrature to yield the total uncertainty.

$p_{\mathrm{T}}^{\mathrm{t\bar{t}}}$ bin range [GeV]	Bin centre [GeV]	$1/\sigma\, \mathrm{d}\sigma/\mathrm{d}p_{\mathrm{T}}^{\mathrm{t\bar{t}}}$ [GeV^{-1}]	Stat. [%]	Syst. [%]	Total [%]
$[0, 30)$	4.50	1.43×10^{-2}	0.8	6.1	6.1
$[30, 80)$	51.50	6.90×10^{-3}	1.0	4.7	4.8
$[80, 170)$	118.50	1.91×10^{-3}	1.1	5.4	5.5
$[170, 300)$	223.50	3.47×10^{-4}	2.1	4.7	5.1
$y_{\mathrm{t\bar{t}}}$ bin range	Bin centre	$1/\sigma\, \mathrm{d}\sigma/\mathrm{d}y_{\mathrm{t\bar{t}}}$	Stat. [%]	Syst. [%]	Total [%]
$[-2.5, -1.5)$	-1.93	4.71×10^{-2}	4.0	6.6	7.7
$[-1.5, -1.0)$	-1.24	1.82×10^{-1}	1.8	1.5	2.3
$[-1.0, -0.5)$	-0.76	3.09×10^{-1}	1.4	1.5	2.1
$[-0.5, 0.0)$	-0.31	4.18×10^{-1}	1.2	1.7	2.1
$[0.0, 0.5)$	0.29	4.09×10^{-1}	1.2	1.2	1.7
$[0.5, 1.0)$	0.76	3.15×10^{-1}	1.5	1.3	2.0
$[1.0, 1.5)$	1.24	1.79×10^{-1}	1.8	1.8	2.6
$[1.5, 2.5)$	1.93	4.59×10^{-2}	4.0	6.0	7.2
$m_{\mathrm{t\bar{t}}}$ bin range [GeV]	Bin centre [GeV]	$1/\sigma\, \mathrm{d}\sigma/\mathrm{d}m_{\mathrm{t\bar{t}}}$ [GeV^{-1}]	Stat. [%]	Syst. [%]	Total [%]
$[340, 380)$	354.50	4.14×10^{-3}	3.0	8.6	9.1
$[380, 470)$	428.50	4.50×10^{-3}	1.7	5.3	5.6
$[470, 620)$	537.50	1.95×10^{-3}	1.8	2.9	3.4
$[620, 820)$	705.50	5.25×10^{-4}	2.8	3.2	4.2
$[820, 1100)$	940.50	1.00×10^{-4}	3.7	7.3	8.2
$[1100, 1600)$	1328.50	7.28×10^{-6}	14.4	28.2	31.6
